# Arginine is an epigenetic regulator targeting TEAD4 to modulate OXPHOS in prostate cancer cells

**DOI:** 10.1038/s41467-021-22652-9

**Published:** 2021-04-23

**Authors:** Chia-Lin Chen, Sheng-Chieh Hsu, Tan-Ya Chung, Cheng-Ying Chu, Hung-Jung Wang, Pei-Wen Hsiao, Shauh-Der Yeh, David K. Ann, Yun Yen, Hsing-Jien Kung

**Affiliations:** 1 Institute of Molecular and Genomic Medicine, National Health Research Institutes, Zhunan, Miaoli County Taiwan; 2 Institute of Biotechnology, National Tsing-Hua University, Hsinchu, Taiwan; 3 Institute of Cellular and System Medicine, National Health Research Institutes, Zhunan, Miaoli County Taiwan; 4 Research Center of Cancer Translational Medicine, Taipei Medical University, Taipei, Taiwan; 5 Institute of Medical Sciences, Tzu Chi University, Hualien City, Taiwan; 6 Agricultural Biotechnology Research Center, Academia Sinica, Taipei, Taiwan; 7 Department of Urology and Oncology, Taipei Medical University Hospital, Taipei, Taiwan; 8 Department of Urology, School of Medicine, College of Medicine, Taipei Medical University, Taipei, Taiwan; 9 Department of Diabetes and Metabolic Diseases Research, Irell & Manella Graduate School of Biological Sciences, Beckman Research Institute, City of Hope, Duarte, CA USA; 10 Ph.D. Program for Cancer Biology and Drug Discovery, College of Medical Science and Technology, Taipei Medical University, Taipei, Taiwan; 11 Department of Biochemistry and Molecular Medicine, Comprehensive Cancer Center, University of California at Davis, Sacramento, CA USA

**Keywords:** Next-generation sequencing, Cancer epigenetics, Cancer metabolism, Targeted therapies

## Abstract

Arginine plays diverse roles in cellular physiology. As a semi-essential amino acid, arginine deprivation has been used to target cancers with arginine synthesis deficiency. Arginine-deprived cancer cells exhibit mitochondrial dysfunction, transcriptional reprogramming and eventual cell death. In this study, we show in prostate cancer cells that arginine acts as an epigenetic regulator to modulate histone acetylation, leading to global upregulation of nuclear-encoded oxidative phosphorylation (OXPHOS) genes. TEAD4 is retained in the nucleus by arginine, enhancing its recruitment to the promoter/enhancer regions of OXPHOS genes and mediating coordinated upregulation in a YAP1-independent but mTOR-dependent manner. Arginine also activates the expression of lysine acetyl-transferases and increases overall levels of acetylated histones and acetyl-CoA, facilitating TEAD4 recruitment. Silencing of TEAD4 suppresses OXPHOS functions and prostate cancer cell growth in vitro and in vivo. Given the strong correlation of TEAD4 expression and prostate carcinogenesis, targeting TEAD4 may be beneficially used to enhance arginine-deprivation therapy and prostate cancer therapy.

## Abstract (web summary)

Alterations in metabolism and amino acid usage are common in cancer cells. Here, the authors show in prostate cancer cells that arginine globally upregulates nuclear-encoded oxidative phosphorylation genes by altering histone acetylation and retaining TEAD4 in the nucleus to transactivate genes.

## Introduction

Arginine and its metabolic products, including nitrogen oxide, polyamines and nucleotides, are vital to cellular physiology, and their dysregulations could contribute to diseases, including cancers^1^. Arginine is one of the three amino acids that can directly activate mammalian target of rapamycin complex 1 (mTORC1), a nutrient-sensing kinase involved in the regulation of cell metabolism, proliferation, and survival^2,3^. Human cells are equipped with the ability to intracellularly synthesize arginine from citrulline and aspartate via two rate-limiting enzymes argininosuccinate synthethase1 (ASS1) and argininosuccinate liase. Interestingly, >70% of the tumor cells have deficiency in this pathway^4^, primarily due to epigenetic suppression of the expression of ASS1^5^. These ASS1-low tumor cells become arginine auxotroph and critically depend on external arginine for survival, which has been exploited therapeutically, using arginine-metabolizing enzymes such as arginase or arginine deiminase (ADI)^6^. Arginase has been clinically approved for the treatment of leukemia and ADI is undergoing clinical trials with excellent safety profile^7–9^.

ADI treatment or arginine starvation effectively kills ASS1-low tumor cells via different mechanisms, including caspase-dependent apoptosis^5,10^ or caspase-independent autophagic death^11,12^. Our studies revealed in prostate and breast cancer cells that arginine starvation induces mitochondrial dysfunction, depletion of mitochondrial metabolites, alteration of mitochondrial morphology, and generation of mitochondrial reactive oxygen species (mitoROS)^4,13,14^ At late phase, the cell killing is associated with nuclear DNA leakage and chromatin autophagy, a consequence of DNA damage and excessive autophagy^4,13,15^. This is accompanied by a coordinated silencing of nuclear-encoded mitochondrial genes, including oxidative phosphorylation (OXPHOS) genes and nucleotide synthesis genes. The mechanism as to how arginine deprivation remodels chromatin and reprograms tumor cell genome remains unclear. Likewise, how arginine deprivation has such a profound effect on mitochondrial functions is not well understood.

Transcriptional enhanced associate domain (TEAD) family of transcription factors are involved in cell proliferation and organ development. Their deregulations are associated with tumor progression and cancer development^16^. TEAD family members, TEAD 1–4, are effectors of several oncogenic pathways and transcriptional activators of oncogenes. They are most recognized as the transcriptional partner of YAP/TAZ in the Hippo pathway^17^. Recent studies indicated that TEAD1 also has HIPPO-independent functions in tumor progression. For instance, TEAD4 was found to induce epithelial-to-mesenchymal transition (EMT) of colon cancer in a Hippo-independent manner^18^. Genome-wide chromatin immunoprecipitation–sequencing (ChIP-seq) analyses of cancer cells reveal the close association of TEAD transcription factor with active histone markers (e.g., acetylated histone H3K27Ac), suggesting its role as a transcriptional activator^19^. While there is considerable evidence implicating TEAD in oncogenesis, little is known about its functions in cancer metabolism. Interestingly, recent work showed that TEAD4, but not other TEAD family members, is located in mitochondrial and involved in the regulation of mitochondria-encoded genes involved in OXPHOS activities^20,21^.

In the present study, we set forth to provide fundamental understanding as to how arginine modulates cellular transcriptional program. Due to its relevance to cancer therapy, we utilized ASS1-low tumor cells, CWR22Rv1 and PC3, addicted to external arginine as our models. We found that arginine is a strong inducer of genome-wide histone acetylation, which is accompanied by an increase of acetyl-coA and mTOR-dependent expression of acetyl-CoA synthesis enzymes, including ACLY and ACSS2. The expression levels of a majority of KATs (lysine acetyl-transferases) are also increased. The genome-wide increase of acetylation, however, is not random and selectively targets certain gene families, including OXPHOS genes. Unbiased screen of motifs associated with acetylation-enriched OXPHOS genes identifies TEAD4 as a potential transcription factor involved in the coordinated transcription. Knockdown of TEAD4 validates its role in the transcription of OXPHOS genes and impairs mitochondrial respiration. In addition, knockdown of TEAD4, inhibits the growth of arginine auxotrophic tumors in vitro and in vivo, similar to ADI-treated samples. Importantly, we found that arginine induces TEAD4’s nuclear retention and enriches its recruitment to enhancers of OXPHOS genes. The recruitment of TEAD4 to the target sites is KAT dependent, suggesting that TEAD4, in conjunction with histone acetylases coordinately activate OXPHOS genes and maintain mitochondrial OXPHOS functions. Together with previous finding demonstrating its role in the transcription of mitochondrial-encoded OXPHOS genes^20,21^, our finding that it also regulated nuclear-encoded OXPHOS genes suggests that TEAD4 is a master regulator of OXPHOS genes and mitochondrial functions in tumor cells. The work also sheds light on how arginine reprograms cellular genome via heightened histone acetylation.

## Results

### Arginine globally modulates metabolic gene expression via histone acetylation

We previously showed that arginine deprivation of tumor cells impairs mitochondrial functions and induces atypical cell death with nuclear DNA leakage and chromatin autophagy^13,14^. It also has a profound effect on tumor metabolism and genes associated with the syntheses of nucleotide, asparagine, proline, serine, and glutamine, as well as mitochondrial functions, in a cell context-dependent manner^4,14,22^. In this study, we wished to explore the molecular basis of the coordinated regulation of the metabolic genes in ASS1-low prostate cancer cells.

**Fig. 1:**
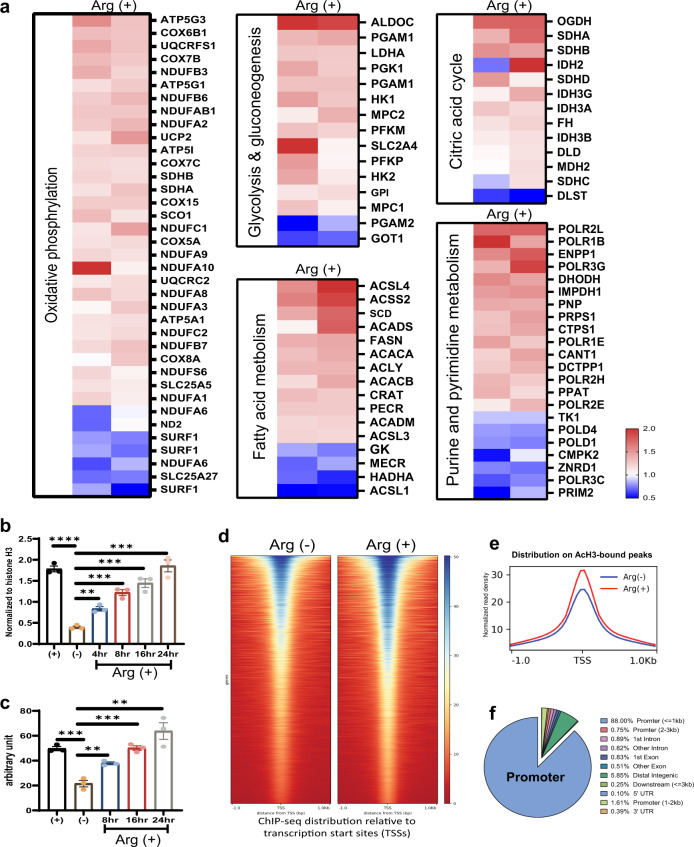
Arginine globally modulates metabolic gene expression via histone acetylation. **a** Heat map of Affymetrix microarray analysis shows arginine (Arg+) globally induced metabolic pathways, including oxidative phosphorylation pathway, glucose, fatty acid, and DNA metabolic pathways in CWR22Rv1 prostate cancer cells. The color key indicates the fold change to control. **b** After starvation overnight, cells were treated with arginine and then harvested at different time points for total acetyl-histone H3 ELISA assay. This data shows that arginine regulated the total level of histone H3 acetylation at different time points. **c** After starvation overnight, cells were treated with arginine and then harvested at different time points for total acetyl-CoA assay. ELISA data show that arginine regulated the total level of acetyl-CoA at different time points. **d** Heat map of acetylated histone H3 ChIP-seq peaks showing that the distribution of peaks are close to transcription star sites (TSS). **e** Overlap of arginine stimulation Arg (+) and arginine deprivation Arg (−) ChIP-seq peaks. **f** Acetylated Histone H3 ChIP-sequencing peaks classified by human genomic annotation (hg38), showing that the peaks of histone acetylation are mainly enriched in the promoter region of genes. Data are presented as mean values ± SEM of independent experiments (*n* = 3 in **b**, **c**). **p < 0.01, ***p < 0.001, ****p < 0.0001, using unpaired two-tailed Student’s *t* test. Source data are provided as a Source data file.

We first examined the metabolic gene expression pattern regulated by the addition of arginine, using CWR22Rv1 (Fig. 1a) and PC3 (Supplementary Fig. 1a) as models. In both cases, arginine stimulation coordinately induced metabolic gene expression, including OXPHOS pathway and pathways associated with glucose metabolism, fatty acid metabolism, and DNA metabolism. This global gene induction pattern implies that arginine may act through epigenetic regulations, including histone acetylation. We then evaluated the level of total histone H3 acetylation after arginine stimulation by enzyme-linked immunosorbent assay (ELISA). As shown in Fig. 1b, the overall histone acetylation as assayed by antibodies against acetyl-histone H3 is much lower in arginine-free media (Arg−) than regular media (Arg+). Addition of arginine increases the level of acetylated histone H3 in a time-dependent manner. We further determined the level of acetyl-CoA, the major source of histone acetylation, after arginine stimulation. As shown in Fig. 1c, the acetyl-CoA level was decreased in arginine-depleted media and restored upon addition of arginine. To study the effect of arginine on histone acetylation at the genome-wide level, we conducted acetyl-histone H3 ChIP-seq with or without arginine stimulation. Based on ChIP-seq data, 14,098 peaks were identified in arginine stimulation group while 11,862 peaks were identified in the arginine-deprivation group (Fig. 1d). These peaks are highly enriched near transcription start sites (TSSs; Fig. 1d, e). With human genomic (hg38) annotation analysis, 88% of peaks are enriched in the gene promoter region (demarcated here as ±1 kb from the TSS, Fig. 1e, f).

### Arginine epigenetically modulates mitochondrial OXPHOS genes

**Fig. 2:**
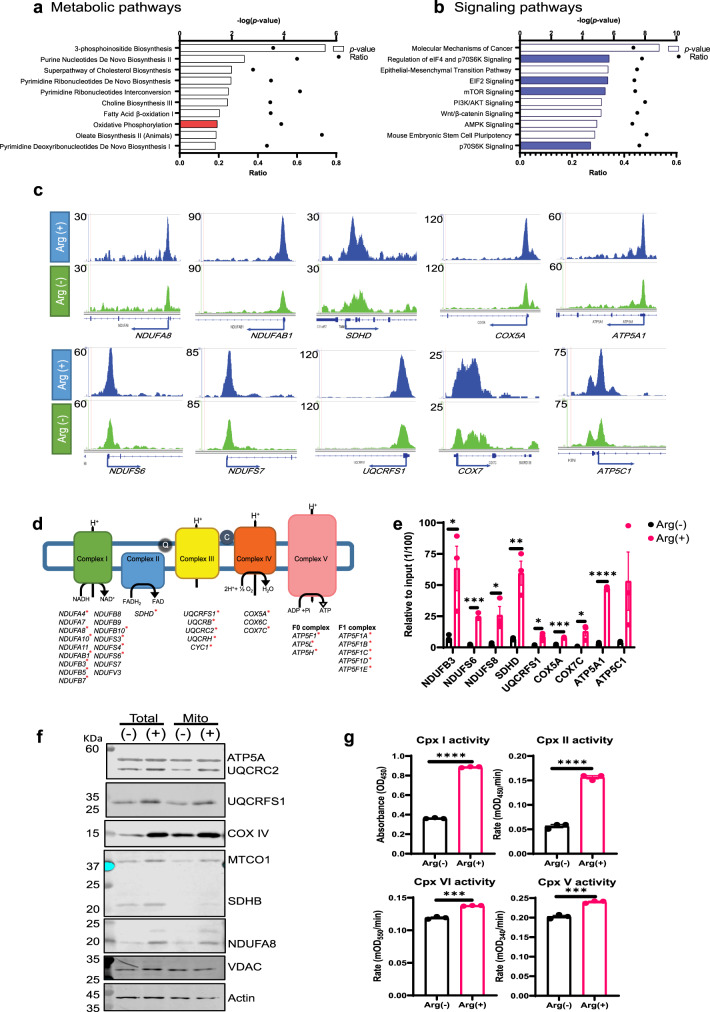
Arginine epigenetically modulates mitochondrial OXPHOS genes. **a** Ingenuity pathway analysis on metabolic pathways shows arginine stimulation epigenetically modulates key metabolic pathways, including plasma membrane biosynthesis, DNA synthesis, and OXPHOS pathways. **b** Ingenuity pathway analysis on cell signaling pathways shows arginine stimulation mainly induced mTOR pathway (highlighted in blue). **c** ChIP-seq distribution for acetylated histone H3 at representative OXPHOS genes loci. **d** The summary list of OXPHOS genes enriched in the arginine-stimulation group based on ChIP-seq data. The upregulated genes in microarray data are marked with an asterisk (*). **e** ChIP-qPCR of histone H3 acetylation confirmed arginine stimulation induced histone acetylation on the promoter region of OXPHOS genes. The value indicated the fold enrichment after arginine stimulation. **f** After starvation, cells were treated with arginine for 24 h and then harvested. Both total lysate and mitochondrial fraction (Mito) were used for immunoblotting. These data show that arginine induced OXPHOS protein expression. **g** After starvation, cells were treated with arginine for 24 h and then harvested for individual complex activity assay. These data show arginine stimulation increased mitochondrial complex activities. Data are presented as mean values ± SEM of independent experiments (*n* = 3 in **e**, **g**). *p < 0.05, **p < 0.01, ***p < 0.001, ****p < 0.0001, using unpaired two-tailed Student’s *t* test. Source data are provided as a Source data file.

We next analyzed the hits, which show higher signal in the arginine-stimulation group for the pathways epigenetically activated by arginine, using ingenuity pathway analysis (IPA) of the ChIP-seq data. Metabolic pathway analysis reveals that the acetylated histone-enriched genes are involved in plasma membrane synthesis, nucleotide biosynthesis, and OXPHOS pathway (red, Fig. 2a). Signaling pathway analysis showed the involvement of cancer-related pathways, including mTOR (highlighted in blue, Fig. 2b), phosphoinositide 3-kinase/AKT, and Wnt/b-catenin pathways. As our previous work showed that arginine deprivation has profound effects on mitochondrial functions, in the present study, we have focused on the OXPHOS genes. We first checked the ChIP-seq distribution for OXPHOS genes. As illustrated in Fig. 2c, the peaks of OXPHOS complex I–V genes are significantly higher and often broader in the arginine-stimulated group (blue) as compared to the control group (green). These peaks are enriched in the promoter region of OXPHOS genes, consistent with the annotation result. Strikingly, >46% (34/73) of the nuclear-encoded OXPHOS genes display heightened histone acetylation in the arginine-stimulation group, suggesting coordinated regulation by arginine (Fig. 2d). ChIP-qPCR (quantitative real-time polymerase chain reaction) of these genes validate the ChIP-seq results at OXPHOS genes (representative samples shown in Fig. 2e). Consistently, 80% of these genes also showed elevated expression at the transcript level (Fig. 1a and starred in Fig. 2d). Both the protein expression levels (Fig. 2f) and the activities (Fig. 2g) of OXPHOS complexes were also increased accordingly. These results suggest that arginine modulates OXPHOS genes and hence the mitochondrial functions via epigenetic regulation.

### Arginine targets TEAD4 to modulate the mitochondrial functions

**Fig. 3:**
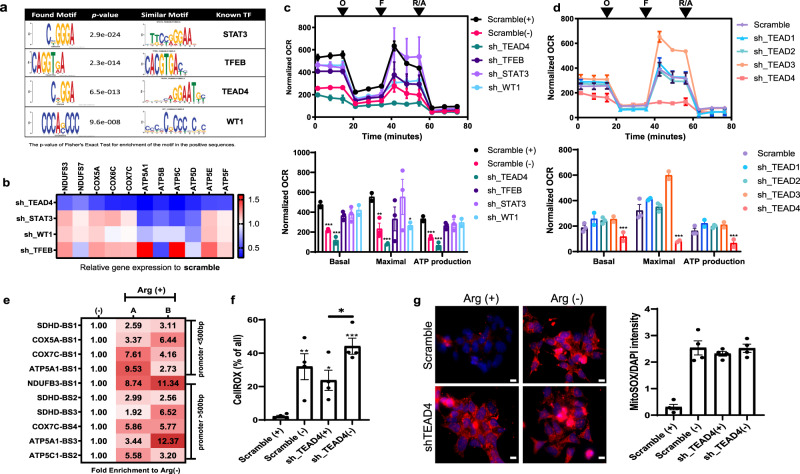
Arginine targets TEAD4 to modulate the mitochondrial functions. **a** The recognition motifs were enriched in ChIP-seq data after arginine stimulation. Top consensus sequences classified by the program DREME, clustered by similarity and order by *p* value. **b** Each candidate was first knocked down by lentivirus transduction for 24 h, following an antibiotic selection for 1 week. Cells were then harvested for real-time PCR analysis of OXPHOS gene expression. The color key indicates the relative gene expression to scramble control. **c** Seahorse assay for mitochondrial respiration activities after silencing each TF candidates via shRNA. Arginine deprivation (−) serves as a negative control. O oligomycin, F FCCP, R/A rotenone/antimycin. The individual shRNA plot is shown in Supplementary Fig. 2c. **d** Each TEAD was first knocked down by lentivirus transduction for 24 h, following an antibiotic selection for 1 week. Seahorse assay for mitochondrial respiration activities. O oligomycin, F FCCP, R/A rotenone/antimycin. **e** ChIP-qPCR data show the binding activity of TEAD4 with (Arg+) or without (Arg−) arginine stimulation. The value indicates the fold enrichment to negative control (Arg−). Lane A is the recruitment profile of TEAD4 onto OXPHOS promoters after 24 h of arginine re-stimulation, whereas lane B is that from cultures grown in arginine-supplemented media for a long time. TEAD4 binding sites (BS) of OXPHOS promoters are shown in Supplementary Fig. 2b. **f** Advance image cytometer analysis shows that silencing of TEAD4 increased cellular ROS production after arginine deprivation for 48 h. **g** Scramble or shTEAD4 cells were first incubated with arginine-free media for 48 h and then incubating with MitoSOX for 15 min as described by the manufacturer. Immunofluorescent staining shows that silencing of TEAD4 increased mitochondrial ROS (MitoSOX) after arginine deprivation (all groups *p* value < 0.0001) (scale bar = 10 μm). Data are presented as mean values ± SEM of independent experiments (*n* = 3 in **b**–**e**, *n* = 4 in **f**, **g**). *p < 0.05, **p < 0.01, ***p < 0.001, using unpaired two-tailed Student’s *t* test. Source data are provided as a Source data file.

The above results showed that arginine had a global effect on histone acetylation; yet, not all genes exhibited enhanced H3 acetylation and not all genes with heightened H3 acetylation were upregulated. We postulated that the coordinated upregulation of OXPHOS genes are guided by certain transcription factors regulated by arginine. To identify the transcription factor(s) that mediates OXPHOS gene transcriptional activation, we conducted the motif analysis of ChIP-seq data via discriminative regular expression motif elicitation (DREME). Among the top ranking motifs (Fig. 3a), TEAD4-binding motif drew our attention, as TEAD4 is found to be recruited to many of the promoters of OXPHOS genes based on ENCODE ChIP database (Supplementary Table 1 and Supplementary Fig. 2a) and its motif is frequently present in the OXPHOS gene promoters (Supplementary Fig. 2b). To explore the functional relevance of TEAD4, we monitored the effect of TEAD4 knockdown on the expression of H3 acetylation-enriched OXPHOS genes and the oxygen consumption rate (OCR) functions. Also included as controls are transcription factors STAT3, WT1, and TFEB, whose binding motifs also rank high in the DREME analysis. As shown in Fig. 3b, silencing TEAD4, but not others, significantly downregulated the expression of OXPHOS genes. Similar results were observed in PC3 cells, indicating it is not a peculiarity of CWR22Rv1 cells (Supplementary Fig. 1d, e). Furthermore, in seahorse experiment, knockdown of TEAD4 has the most devastating effects on OCR in both cell types (Fig. 3c and Supplementary Fig. 2c). It is noteworthy that silencing other three members of the TEA domain family did not significantly suppress the mitochondrial respiration activity (Fig. 3d), indicating a unique role of TEAD4 in regulating OXPHOS functions. ChIP-PCR experiments presented in Fig. 3e confirmed the enrichment of TEAD4 binding to the representative OXPHOS gene promoters (Supplementary Fig. 2b) upon arginine addition. These data together uncover TEAD4 as an arginine-inducible regulator of OXPHOS genes in tumor cells. Interestingly, we found that, in RWPE1, the immortalized prostate epithelial cell, which expresses a high level of ASS1 and is non-arginine auxotrophic, silencing TEAD4 in RWPE1 cell did not significantly affect the OXPHOS expression either at the transcript or at the protein level, suggesting that the requirement of TEAD4 to activate nuclear OXPHOS genes is tumor selective (Supplementary Fig. 2d, e), which renders TEAD4 as a potential therapeutic target for prostate cancer. That said, we noted that, in mouse embryo cells, TEAD4 is also involved in the transcription of mitochondrial-encoded OXPHOS genes^20,21^. Thus, we cannot exclude the possibility of TEAD4’s requirement for mitochondrial activities in certain non-malignant cell types.

One major consequence of the dysregulated OXPHOS function is the generation of ROS. We previously showed that arginine deprivation induces excessive ROS, eventually leading to DNA damage and cell death^13^ (Supplementary Fig. 2f). We thus asked whether TEAD4 is also involved in the regulation of ROS production by staining with cellular ROS dye (CellROX). As shown in Fig. 3f for CWR22Rv1 cells and in Supplementary Fig. 1f for PC3 cells, arginine deprivation significantly increased the level of cellular ROS. Silencing of TEAD4 with short hairpin RNA (shRNA) alone in the presence of arginine recapitulates the ROS increase. Immunofluorescence staining with mitoROS dye, MitoSOX, further supports this notion (Fig. 3g). Based on transmission electron microscopic data, the mitochondrial ultrastructure was significantly altered upon TEAD4 knockout (KO) or arginine deprivation, and the phenotypes become even more severe in TEAD4 KO plus arginine deprivation (Supplementary Fig. 2g). These results suggest that TEAD4 is a key regulator of OXPHOS genes and mitochondrial functions.

### Arginine mediates nuclear retention of TEAD4

**Fig. 4:**
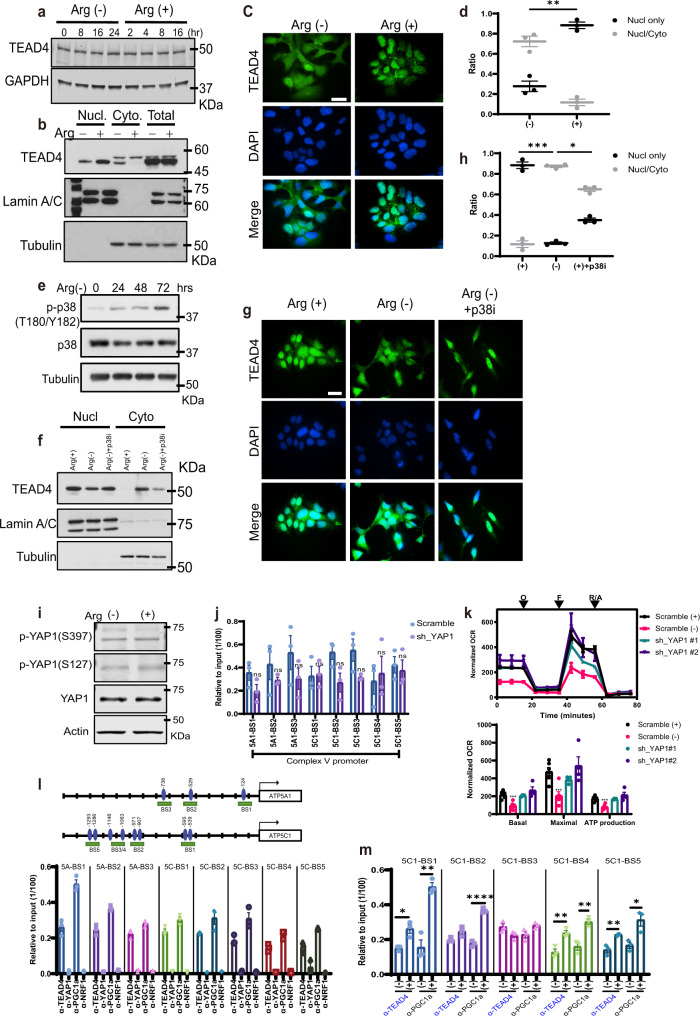
Arginine mediated nuclear retention of TEAD4. **a** Cells were first incubated in arginine-free media and harvested at different time points as indicated. Fresh arginine was then added into media and cells were harvested at different time points as indicated. Western blotting shows that arginine (Arg) did not affect TEAD4 protein level, but affected the ratio of nucleus and cytosol fractions (**b**). **c** Cells were first incubated in arginine-free media for 24 h. Arginine was then added into media for 24 h. Immunofluorescent staining of TEAD4 shows cytosolic translocation after arginine deprivation (scale bar = 20 μm). The quantification of immunofluorescent staining is shown in **d**. **e** Cells were incubated in arginine-free media and harvested at different time points as indicated. Western blotting shows that arginine depletion induced p38 phosphorylation in a time-dependent manner, which is consistent with TEAD4 cytosol translocation (Supplementary Fig. 3a). **f** Cells were incubated in arginine-free media with or without p38 inhibitor (p38i) SB203580 treatment for 24 h. Western blotting shows that inhibition of p38 activation prevented TEAD4 cytosolic translocation upon arginine deprivation. The total TEAD4 expression is shown in Supplementary Fig. 3b. **g** Immunofluorescent staining of TEAD4 shows that inhibition of p38 activation impeded TEAD4 cytosolic translocation upon arginine deprivation (scale bar = 20 μm). The quantification of immunofluorescent staining is shown in (**h**). **i** After starvation overnight, cells were treated with arginine for 24 h and then harvested for immunoblotting. Western blotting shows that arginine did not affect the phosphorylations of YAP1. **j** YAP1 was knocked down via shRNA lentivirus infection for 24 h, following an antibiotic selection for 1 week. ChIP-qPCR data show that silencing of YAP1 did not significantly affect the binding activity of TEAD4 on OXPHOS promoter region. **k** Cells were transduced with two individual YAP1 shRNAs for 24 h, following an antibiotic selection for 1 week. Seahorse assay for mitochondrial respiration activities after silencing of YAP1 via two individual shRNA clones. O oligomycin, F FCCP, R/A rotenone/antimycin. **l** ChIP-qPCR data show the binding activity of TEAD4, YAP1, PGC1a, and NRF1 on OXPHOS promoters. **m** ChIP-qPCR data show that the presence (+) or absence (−) of arginine regulates the recruitment of TEAD4 and PGC1a on OXPHOS promoters. Data are presented as mean value ± SEM of independent experiments (*n* = 3 in **d**, **h**, **j**, **l**, **m**; *n* = 4 in **k**). *p < 0.05, **p < 0.01, ***p < 0.001, ****p < 0.0001, ns not significant, using unpaired two-tailed Student’s *t* test. Source data are provided as a Source data file.

To understand the mechanism whereby arginine induces the recruitment of TEAD4 to its chromatin-binding site, we first studied the TEAD4 expression level and found that there is no significant difference in the presence or absence of arginine (Fig. 4a). Interestingly, we observed that arginine regulates the cyto-nuclear translocation of TEAD4 (Fig. 4b–d). In the absence of arginine, there is a higher level of TEAD4 localized in the cytosol; addition of arginine compacts TEAD4s into the nucleus. It has been shown that activation of p38 pathway induces TEAD4 cytosolic translocation through direct interaction^23^. We thus investigated whether p38 pathway is involved in arginine targeting TEAD4. As shown in Fig. 4e and Supplementary Fig. 3a, arginine depletion induced p38 phosphorylation, likely due to endoplasmic reticulum stress^4^. As shown in Fig. 4f–h and Supplementary Fig. 3b, inhibition of p38 activation via p38 inhibitor (SB203580) treatment prevented TEAD4 cytosolic translocation after arginine deprivation. These results suggest that arginine suppresses p38 activation to retain TEAD4 in the nucleus.

YAP1 (yes-associated protein 1) is the major effector of Hippo signaling pathway, which has been widely considered as a major coactivator of TEAD proteins. To evaluate whether arginine targets TEAD4 through YAP1 signaling pathway, we first checked YAP phosphorylation on Serine-397, which destabilizes YAP1, as well as on Serine-127, which retains YAP1 in the cytosol. As shown in Fig. 4i, arginine stimulation did not affect either phosphorylation of YAP1 or the total protein level. We further knocked down YAP1 expression by two individual shRNA clones, which did not affect the total TEAD4 protein level (Supplementary Fig. 3c). Furthermore, silencing YAP1 did not significantly affect the recruitment of TEAD4 onto the OXPHOS promoters (Fig. 4j). Functionally, silencing of YAP1 did not significantly interfere with the mitochondrial respiration activity (Fig. 4k). These results suggest that TEAD4-mediated mitochondrial functions and its recruitment to OXPHOS promoter region in the presence of arginine is YAP1 independent.

As a transcription factor, TEAD4 requires a coactivator for full activity. Given that YAP1 is not involved, we asked whether other transcriptional coactivators may be involved. PGC1a, serving as a coactivator for NRF1, plays a key role in mitochondrial biosynthesis. Using ATP5A1 and ATP5C1 promoters that contain multiple TEAD4-binding sites, we tested the recruitment of PGC1a to these sites (Fig. 4l). We found by ChIP-qPCR that PGC1a intensely bound to TEAD4-binding sites and its recruitment was generally stimulated by arginine (Fig. 4m). Furthermore, co-immunoprecipitation result showed the physical association of endogenous TEAD4 with PGC-1a (Supplementary Fig. 3d). In the same set of experiments, we also monitored the recruitment of NRF1 to these sites. NRF1, a binding partner of PGC-1a, was not recruited to these sites, indicating that the binding of PGC1a to TEAD4 is not mediated by NRF1 on these sites (Fig. 4l). PGC1a is known to be activated by AMPK. To study whether AMPK is involved in catalyzing the interaction between PGC1a and TEAD4, we modulated AMPK activities by AMPK activator, metformin, and selective AMPK inhibitor, dorsomorphin. The results showed that neither activation nor inhibition of AMPK affects TEAD4 protein expression level (Supplementary Fig. 3e), nuclear retention (Supplementary Fig. 3f), and the interaction with PGC1a (Supplementary Fig. 3g). Thus, in our system, AMPK does not seem to play a role in the regulation of TEAD4. These data suggest that PGC1a is involved in the activation of OXPHOS genes. There are likely other coactivators involved, which have yet to be discovered. NRF1 may also be involved in this process but utilizes its own binding sites present in the promoters of OXPHOS genes.

### Arginine modulates OXPHOS pathway and TEAD4 recruitment is mTOR dependent

**Fig. 5:**
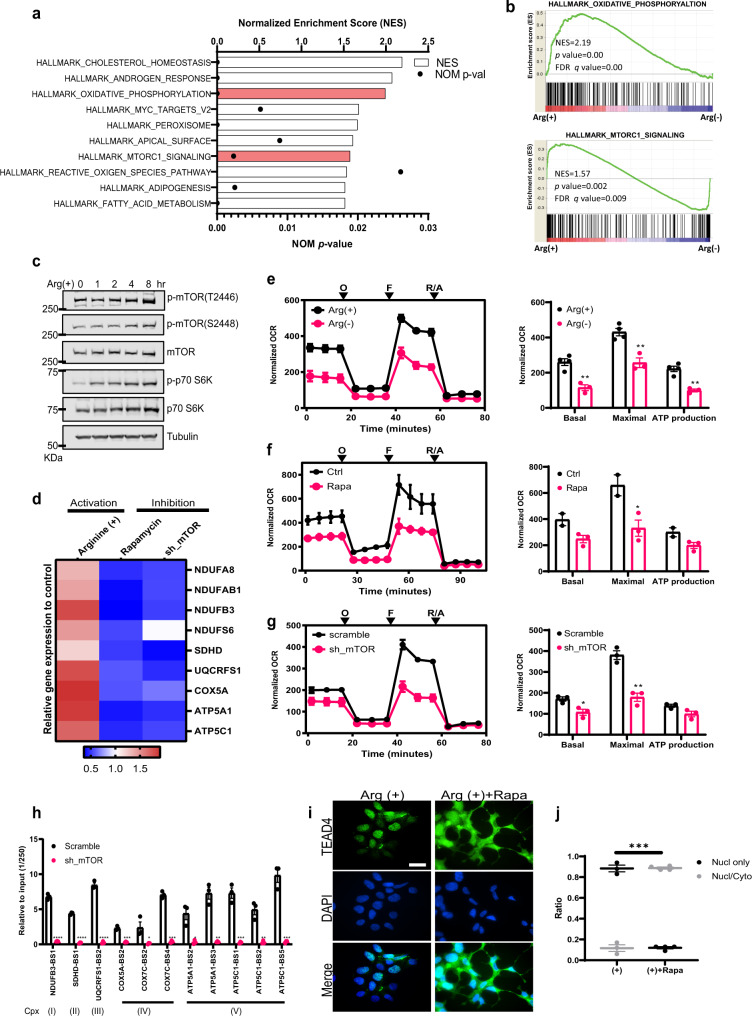
Arginine modulates OXPHOS pathway and TEAD4 recruitment is mTOR dependent. **a** Gene set enrichment assay (GSEA, v4.1.0) of microarray data was performed using hallmark gene set collection. Normalized enrichment scores (NES) for pathways indicate the significant difference in the arginine-stimulation group. **b** GSEA shows that the genes associated with oxidative phosphorylation (upper panel) and mTORC1 pathway (lower panel) are enriched in the arginine-stimulation Arg (+) group. **c** After starvation overnight, cells were treated with arginine and harvested at different time points as indicated. Western blotting shows that arginine stimulation induced the phosphorylation of mTORC1 (T2446 and S2448) and its downstream target, phosphor-p70 S6K. **d** qPCR analysis of OXPHOS gene expression after mTOR activation by arginine stimulation and mTOR inactivation by rapamycin treatment (24 h) or shRNA knockdown. The color key indicates the relative gene expression to vehicle or scramble control. **e**–**g** Seahorse assay shows that activation of mTOR by 24-h arginine stimulation (**e**) increases the mitochondrial respiration activity. By contrast, inactivation of mTOR by 24-h rapamycin treatment (**f**) or shRNA knockdown (**g**) suppressed the mitochondrial respiration activity. O oligomycin, F FCCP, R/A rotenone/antimycin. **h** ChIP-qPCR of TEAD4 on OXPHOS gene promoter region shows that silencing of mTOR disrupted the TEAD4 recruitment after arginine stimulation. **i** Immunofluorescent staining of TEAD4 shows that inhibition of mTOR via rapamycin treatment induced TEAD4 cytosolic translocation (scale bar = 20 μm). The quantification of immunofluorescent staining is shown in **j**. Data are presented as mean value ± SEM of independent experiments (*n* = 3 in **d**–**h**, **j**). *p < 0.05, **p < 0.01, ***p < 0.001, ****p < 0.0001, using unpaired two-tailed Student’s *t* test. Source data are provided as a Source data file.

As described above, we showed that arginine induces TEAD4 to modulate the OXPHOS gene expression, and this process is associated with acetylation but independent of YAP1. We next investigated whether other signal pathways are involved. As shown in Fig. 5a, b for CWR22Rv1 and in Supplementary Fig. 1b, c for PC3, when the gene signature of arginine-stimulated cells was analyzed by microarray, both OXPHOS and mTOR pathways were affected, consistent with the H3 acetylation ChIP-seq data. Arginine is a known activator of mTOR^24^. We wished to study mTOR’s involvement in arginine-mediated histone acetylation and TEAD4 recruitment onto OXPHOS genes. As shown in Fig. 5c, arginine induced the activating phosphorylation of mTOR on both T2446 and S2448 sites at 8 h post arginine addition, as well as the phosphorylation of its downstream effector, p70 S6K. We then investigated whether representative OXPHOS gene expression is affected by mTOR signaling pathway via rapamycin treatment or mTOR shRNA. As shown in Fig. 5d, the majority of the OXPHOS genes tested are inhibited by either treatment. Likewise, these treatments impaired OCR (Fig. 5e–g). Importantly, silencing of mTOR disrupted the TEAD4 recruitment on OXPHOS promoter region after arginine stimulation (Fig. 5h). Additionally, similar to arginine-deprivation condition, inactivation of mTORC1 pathway by rapamycin treatment induced TEAD4 cytosolic translocation (Fig. 5i, j). These data together suggest that arginine targets TEAD4 to modulate the mitochondrial function through mTOR pathway.

### Arginine activates mTOR pathway to regulate the level of acetyl-CoA and modulate histone acetylation

**Fig. 6:**
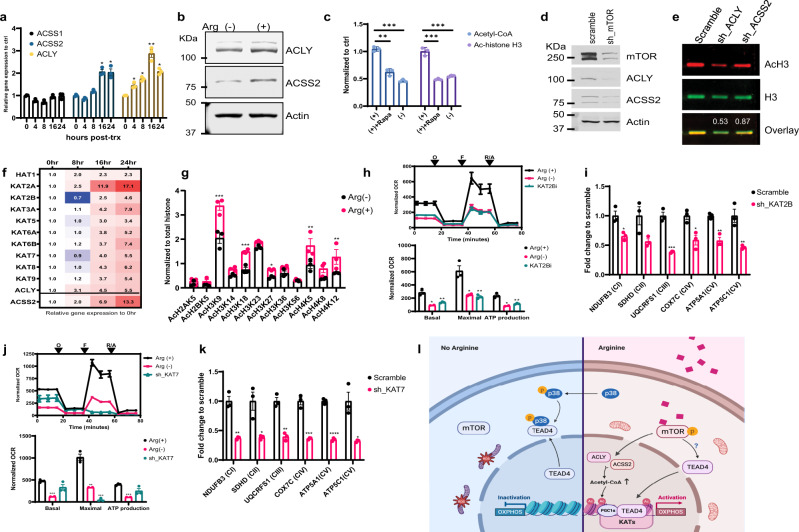
Arginine activates mTOR pathway to regulate the level of acetyl-CoA and modulate histone acetylation. **a** After starvation overnight, cells were treated with arginine and harvested at different time points as indicated. qPCR analysis shows the gene expression of ACLY and ACSS2, but not of ACSS1, were upregulated after arginine stimulation in a time-dependent manner. **b** After arginine starvation, cells were treated with arginine for 24 h. Western blotting shows that arginine stimulation (+) induced the protein expression of ACLY and ACSS2. **c** Cells were grown either in regular media supplemented with rapamycin or in arginine-free media for 24 h. ELISA data show that inhibition of mTOR activity via rapamycin treatment or arginine deprivation decreased the level of acetyl-CoA (blue) and histone H3 acetylation (purple). **d** Cells were first transduced with mTOR shRNA for 24 h, following an antibiotic selection for 1 week. Western blotting shows that the protein expression of ACLY and ACSS2 were downregulated after silencing of mTOR. **e** Cells were first transduced with ACLY or ACSS2 shRNA for 24 h, following an antibiotic selection for 1 week. Western blotting data show that the ratio of acetyl-histone H3 was decreased after silencing of ACLY or ACSS2. **f** After starvation overnight, cells were treated with arginine and harvested at different time points as indicated. qPCR analysis shows that arginine stimulation globally induced the histone acetyltransferase (HAT) expression. The color key indicates the relative gene expression to vehicle control (at time 0 h). The expression of ACLY and ACSS2 serves as an internal positive control. **g** After arginine starvation, cells were treated with arginine for 24 h. ELISA data show that arginine stimulation induced the histone H3 acetylation, specifically on lysine residues 9, 18, and 27, as well as H4 acetylation on lysine residues 5 and 12. **h** Cells were grown in regular media treated with KAT2B inhibitor (KAT2Bi), Garcinol, or in arginine-free media for 24 h. Seahorse data show that inhibition of KAT2B significantly suppressed the mitochondrial respiration activities. O oligomycin, F FCCP, R/A rotenone/antimycin. **i** Cells were first transduced with KAT2B shRNA for 24 h, following an antibiotic selection for 1 week. ChIP-qPCR shows the TEAD4 recruitment on OXPHOS promoter regions after silencing of KAT2B. **j** Seahorse data show that silencing of KAT7 significantly suppressed the mitochondrial respiration activities. O oligomycin, F FCCP, R/A rotenone/antimycin. **k** Cells were first transduced with KAT7 shRNA for 24 h, following an antibiotic selection for 1 week. ChIP-qPCR shows the TEAD4 recruitment on OXPHOS promoter regions after silencing of KAT7. **l** Diagram of hypothetical model. Data are presented as mean value ± SEM of independent experiments (*n* = 3 in **a**, **c**, **f**–**k**). *p < 0.05, **p < 0.01, ***p < 0.001, ****p < 0.0001, using unpaired two-tailed Student’s *t* test. Source data are provided as a Source data file.

The above results showed that arginine-induced, TEAD4-dependent activation of OXPHOS genes is associated with histone acetylation and depends on mTOR signaling. To study the relationship between arginine-induced mTOR signaling and histone acetylation, we examined the arginine regulation of key acetyl-CoA synthetic enzymes, ACLY, ACSS1 (mitochondria-associated), and ACSS2 (nucleus-associated). As shown in Fig. 6a, b, arginine stimulation upregulated the expression of ACLY and ACSS2 both at the transcript and protein level. By contrast, ACSS1 is not affected. We then asked whether mTOR signal pathway is involved in these regulations. First, inactivation of mTOR activity via rapamycin treatment partially reduced the level of acetyl-CoA and histone H3 acetylation (Fig. 6c). Second, silencing of mTOR via shRNA downregulated the protein level of ACLY and ACSS2 (Fig. 6d) Finally, silencing of ACLY and ACSS2 via shRNA reduced the total level of histone H3 acetylation (Fig. 6e). These data suggest that arginine-mediated OXPHOS genes activation depends on mTOR activation, through the increase of acetyl-CoA production and upregulation of acetyl-CoA synthetic enzymes. The expression levels of ACLY and ACSS2, while depending on mTOR, do not correlate with AMPK activities (Supplementary Fig. 3h).

### Arginine upregulates KATs to enhance histone acetylation and TEAD4 activation of OXPHOS genes

The last missing link between arginine stimulation and histone acetylation is the family of lysine acetylases (KATs). We found that there is a general induction of the expression of all KATs, upon arginine stimulation (Fig. 6f), with the consequent increase of histone acetylation at varying degrees on various lysine residues on H3 and H4 (Fig. 6g). Acetylation on H2A and H2B is less affected. Most notable are the increases of histone H3 acetylation on lysine residues 9, 18, and 27 and those of histone H4 on lysine residues 5 and 12. It thus appears multiple KATs are involved in the induction of histone acetylation upon arginine stimulation. To demonstrate that these KATs are involved in OXPHOS activation and TEAD4 recruitment, we selected two KATs, KAT2B and KAT7, which respectively catalyze histone H3 and H4 for further analyses. KAT2B catalyzes the acetylation of H3K9, H3K18, and H3K27, while KAT7 acetylates H4K5 and H4K12. Inhibition of either of these two KATs, especially KAT2B, significantly reduced the level of mitochondrial respiration activity (Figs. 6h, j) as well as TEAD4 recruitment to OXPHOS promoters (Fig. 6i, k).

In summary (Fig. 6l), our data provide the following model for arginine-mediated activation of OXPHOS genes: (1) arginine activates mTOR to upregulate ACLY and ACSS2 for enhanced production of acetyl-CoA; (2) arginine upregulates the expression of KATs, which in combination with increased acetyl-CoA enhances overall histone acetylation; (3) arginine prevents p38 activation to retain TEAD4 in the nucleus, which together with PCG1a is recruited to OXPHOS promoters. The overall consequence is the coordinated activation of OXPHOS genes.

### TEAD4 could be a potential target for prostate cancer therapy

**Fig. 7:**
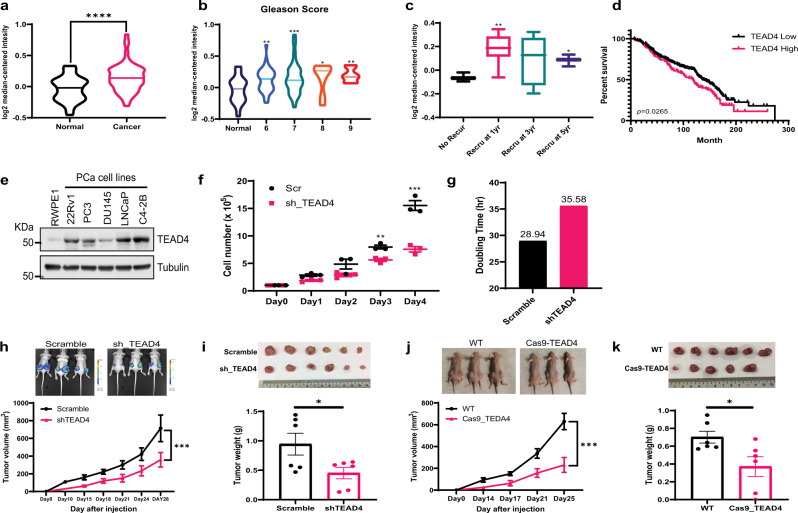
TEAD4 could be a potential target for cancer therapy. **a** TEAD4 expression in Oncomine (www.oncomine.org) Taylor prostate cancer database (*n* for normal = 29, *n* for cancer = 131, the center line indicates median, ****p < 0.0001). **b** Association of TEAD4 expression with Gleason score in Oncomine (www.oncomine.org) Taylor prostate cancer database (*n* for normal = 25, *n* for Grade 6 = 41, *n* for Grade 7 = 74, *n* for Grade 8 = 8, *n* for Grade 9 = 7, the center line indicates the median, *p < 0.05, **p < 0.01, ***p < 0.001). **c** Correlation of TEAD4 expression with tumor recurrence in Oncomine (www.oncomine.org) Taylor prostate cancer database (*n* for no recurrence = 3, *n* for recurrence at first year = 9, *n* for recurrence at third year = 11, *n* for recurrence at fifth year = 3, whiskers = min to max, bounds of box = lower quartile and upper quartile, the center line indicates the median, *p < 0.05, **p < 0.01). **d** Kaplan–Meier survival plot shows the correlation of TEAD4 expression with survival rate in Oncomine (www.oncomine.org) Setlur prostate cancer database (*n* for low expression = 243, *n* for high expression = 119). **e** TEAD4 expression in immortalized prostate cell line, RWPE1, and prostate cancer cell lines, CWR22Rv1, PC3, DU145, LNCaP, and C4-2B. **f** 1 × 10^5^ of scrambled shTEAD4 cells were plated in 24-well plate. Cell growth after silencing of TEAD4 by shRNA in CWR22Rv1 cells. **g** Doubling time after silencing of TEAD4 by shRNA in CWR22Rv1 cells. **h** Tumor growth curve of silencing of TEAD4 by shRNA in CWR22Rv1 cells and tumor image by IVIS scan at endpoint. **i** Isolated tumor image and weight at endpoint. **j** Tumor growth curve of knockout of TEAD4 by CRISPR-Cas9 in CWR22Rv1 cells and tumor image at endpoint. **k** Isolated tumor image and weight at endpoint. Data are presented as mean value ± SEM of independent experiments (*n* = 3 in **f**, *n* = 6 in **h**–**k**). *p < 0.05, **p < 0.01, ***p < 0.001, ****p < 0.0001, using unpaired two-tailed Student’s *t* test. Source data are provided as a Source data file.

Finally, to analyze the clinical relevance of TEAD4 in prostate carcinogenesis, the Oncomine™ Platform (Thermo Fisher, Ann Arbor, MI) was used for analysis and visualization. As shown in Fig. 7a, TEAD4 is highly expressed in cancer tissue when compared to normal tissue. Furthermore, TEAD4 is also associated with the disease progression predictor of prostate cancer, Gleason score (Fig. 7b). Importantly, TEAD4 is associated with tumor recurrence and survival rate. As shown in Fig. 7c, those patients with recurrent cancer at first year show the highest TEAD4 expression. Moreover, those patients with higher TEAD4 expression show lower survival rate (Fig. 7d). It is noteworthy that the correlation of the expression of other TEAD members (TEAD1-3) with prostate cancer progression are not as strong (Supplementary Fig. 4a). In addition, YAP1 expression is not positively correlated with TEAD4, supporting a YAP1-independent function of TEAD4 in prostate carcinogenesis (Supplementary Fig. 4b). The data collectively point to TEAD4 as a progression factor for prostate cancer.

To experimentally test this possibility, we determined the expression levels of TEAD4 in various prostate cell lines. As shown in Fig. 7e, TEAD4 is highly expressed in various prostate cancer cell lines but not in normal prostate cell line, RWPE1 cells. In vitro, silencing of TEAD4 in prostate cancer cells significantly inhibited cell growth (Fig. 7f for CWR22Rv1 and Supplementary Fig. 1g for PC3) and increased the cell doubling time (Fig. 7g). In vivo, silencing of TEAD4 by shRNA (Fig. 7h, i and Supplementary Fig. 4c) or CRISPR-Cas9 KO of both TEAD4 alleles (Fig. 7j, k and Supplementary Fig. 4d) significantly suppressed the tumor growth. Moreover, we observed the reduction of pan-OXPHOS protein and proliferation marker, Ki67, in tumors, while the expression levels of cyclin-dependent kinase inhibitors, p21 and p27, were increased. (Supplementary Fig. 4e). These data provided a proof-of-principle experiment for TEAD4 as a potential therapeutic target for prostate cancer.

## Discussion

In previous studies, we showed that arginine starvation has a detrimental effects on mitochondria and genome integrity of tumor cells, leading to nuclear DNA leakage and chromatin autophagy with eventual cell death^4,13,14^. Here, we present evidence to indicate that arginine is a potent epigenetic regulator, especially for mitochondria OXPHOS genes, in tumor cells addicted to external arginine. A significant number of tumor cells of various cancer types are deficient in intracellular arginine synthesis due to the repressed expression of ASS1^25^. As a consequence, they are arginine auxotroph and require external arginine for growth and survival. Addition of arginine to these cells rapidly remodels chromatin as evidenced by the increased level of genome-wide acetylation of histones and upregulation of metabolic genes and DNA repair genes. Ingenuity pathway analyses (IPAs) of ChIP-seq data of acetylated histones (AcH3) and transcriptome profiling assay upon arginine addition yield converging results with notably the upregulation of the mitochondrial OXPHOS and fatty acid synthesis pathways. In the present study, we have focused on the mitochondrial OXPHOS pathway with an aim to provide a better understanding of the mechanisms associated with their activation.

The increased level of histone acetylation is consistent with the elevated level of acetyl-CoA as well as the heightened expression of ACCS2, ACLY, and the majority of KAT enzymes. Many of these enzymes are under the regulatory control of mTORC1^26–28^, which is activated by arginine. We found acetylated histones (as detected by AcH3) is enriched primarily on the TSS and as such they “marked” the genes upregulated by arginine. Forty-six percent of the nuclear-encoded OXPHOS genes are enriched in H3 acetylation, which correlate well with the OXPHOS genes upregulated by arginine based on transcriptome genomic analysis (Fig. 2d). While KATs are responsible for histone acetylation, the specificities of acetylation at OXPHOS genes are likely to be dictated by transcription factors. Motif analyses of the promoters of AcH3-enriched genes identify the binding sites of TEAD4, STAT3, WT1, and TFEB. shRNA knockdown experiments targeting these transcription factors showed that TEAD4 is the most critical in activating the expression of OXPHOS genes and its recruitment to the OXPHOS promoter target sites is enhanced by arginine (Fig. 3e). TEAD4 is a member of the TEAD family of transcription factors, known to be partners of YAP, an oncogene involved in cell contact and tissue growth^29^. TEAD4 has additional specialized functions and is involved in trophectoderm development^20^. Recent studies reported that TEAD4 can be found in mitochondria and is involved in transcriptional activation of mitochondria-encoded OXPHOS genes^21^. In this regard, TEAD4 is unique among the TEAD4 family members. In resonance, we found that TEAD4, but not other members, is involved in the activation of nuclear-encoded OXPHOS genes, suggesting its role as a master regulator of OXPHOS genes. When the AcH3 acetylation ChIP-seq data and TEAD4-ChIP data from ENCODE database (https://www.encodeproject.org/) are compared, we found that ~66.37% of AcH3-ChIP hits overlapped with those of TEAD4-ChIP. Detailed analysis of the pathways of the overlapped hits via gProfiler revealed that TEAD4 is responsible for the activation of not only the mitochondrial genes but also other metabolic genes as well. This indicates that TEAD has pleiotropic effects on cell metabolism and may explain its potency as an oncogene.

How does arginine activate TEAD4 and enhance its recruitment or OXPHOS genes? We do not exactly know. We found that arginine enhances TEAD4’s nuclear translocation. This process is YAP independent, as YAP is not found to be associated with the OXPHOS promoters and that YAP knockdown by shRNA had no effect on TEAD4 recruitment to OXPHOS promoter or mitochondrial respiratory function (Fig. 4i, j). It has recently been reported that p38 plays an important role in cyto-nuclear translocation of TEAD4. When p38, as stress response kinase, is activated or phosphorylated at Thy180/Tyr182, it translocates TEAD4 from nucleus to cytosol^23^. We showed that p38 is activated upon arginine starvation and stays unphosphorylated in the presence of arginine. Our data suggest that suppression of p38 activity by arginine is in part responsible for TEAD4 retention in the nucleus, although we cannot exclude the involvement of other pathways. One such pathway could be mTORC1. Treatment with rapamycin, mTORC1 inhibitor, also induced the cytosolic translocation of TEAD4 (Fig. 5i), although p38 activation was not involved (data not shown). It is possible that mTOR retains TEAD4 in the nucleus indirectly by increasing its binding affinity toward chromatin sites via enhanced histone acetylation through increased KATs, ACLY, and ACSS2, as well as acetyl-coA. Indeed, knockdown of KATs affected TEAD4 recruitment (Fig. 6k, l). Other possibilities include mTOR-mediated posttranslational modification of TEAD4, which awaits further investigations.

YAP1 is the predominant coactivator of TEAD4^30^. Given that YAP1 is not involved in this case, we looked for other coactivator for TEAD4. Our initial results point to PGC-1a, a nuclear factor known to be involved in mitochondrial functions. PGC-1a serves as a coactivator of NRF1 to upregulate TFAM (mitochondrial transcription factor A) for the transcription of mitochondrial genome^31–33^. It also facilitates mTOR’s interaction with YY1 to form the complex, leading to mitochondrial gene expression^34^. Here, we found PGC-1a to be corecruited with TEAD4 to OXPHOS gene promoters (Fig. 4k, l). This interaction does not appear to depend on NRF1. There are, however, NRF1 sites located near TEAD4-binding sites in several of the OXPHOS promoters, indicating a possible synergism.

TEAD4 has been found to be overexpressed in various types of cancers, including breast cancer^35^, colorectal cancer^18,36^, and melanoma^37^. These studies showed that overexpression of TEAD4 in tumor cells plays a critical role in EMT, metastasis, and patients’ survival rate. In this study, we reported its new found role in prostate cancer development by maintaining OXPHOS functions. While in tumor cells, Warburg effect takes the heavy lifting of metabolic activities from OXPHOS to glycolysis, mitochondrial activity is still required for energy homeostasis and biosynthesis of macromolecules^38,39^. We showed that TEAD4-mediated OXPHOS transcription is critical in making up the OXPHOS activity and preventing excessive ROS production. Our work thus not only provides fundamental understanding of TEAD4’s functions in mitochondrial biology but also uncovers TEAD4 as a potential intervention target for prostate cancer.

## Methods

### Cell lines

Prostate cancer cell lines, including CWR22Rv1(ATCC CRL-250), PC3 (ATCC CRL-1435), LNCaP (ATCC CRL-1740), DU145 (ATCC HTB-81), C4-2B (ATCC CRL-3315), and immortalized prostate cell line, RWPE-1 (ATCC CRL-11609), were grown in RPMI-1640 supplemented with 10% fetal bovine serum and 1% penicillin/streptomycin. For arginine-deprivation condition, RPMI-1640 medium without L-Arginine, L-Leucine, and L-Lysine (US Biological, R8999-03A) supplemented with 10% dialysis fetal bovine serum, 1% penicillin/streptomycin, L-Leucine (50 mg/ml, Sigma), and L-Lysine (40 mg/ml, Sigma) was used. For arginine-stimulation experiment, L-Arginine (200 mg/ml) was freshly added after overnight arginine starvation. Cells were routinely tested for mycoplasma contamination using a PCR-based detection analysis (e-Myco #25233).

### Antibodies

TEAD4 (#ab58310), pan-OXPHOS (#ab110413), and Ki67 (#ab15580) were purchased from Abcam. p-mTOR (T2446) (#09-345) and acetyl-histone H3 (#06-599) were purchased from Millipore. Phosphor-PGC1a (#AF6650) was purchased from R&D. mTOR (#2938), p-mTOR (S2448) (#5536), YAP (#14074), p-YAP (S127) (#13008), p-YAP(S397) (#13619), p38 (#8690), p-p38 (T180/182) (#4511), acetyl-histone H3 (Lys9) (#9649), acetyl-histone H3 (Lys14) (#7627), acetyl-histone H3 (Lys18) (#13998), acetyl-histone H3 (Lys27) (#8173), acetyl-histone H3 (Lys56) (#4243), histone H3 (#4499), acetyl-histone H2A (Lys5) (#2579), acetyl-histone H2B (Lys5) (#12799), histone H2A (#12349), histone H2B (#12364), acetyl-histone H4 (Lys5) (#8647), acetyl-histone H4 (Lys8) (#2594), acetyl-histone H4 (Lys12) (#13944), acetyl-histone H4 (#2935), PGC-1a (#2178), p21 (#2974), and p27 (#83630) were purchased form Cell Signaling. All antibodies were used at a dilution of 1:1000 for immunoblotting and 1:250 for immunofluorescent staining.

### Microarray assay

After arginine-stimulation treatment, the total RNA was extracted from the CWR22Rv1 cells by the RNeasy Mini Kit (Qiagen) according to the manufacturer’s instructions. Microarray gene expression profiling was performed by the Transcriptome Analysis Console software (Thermofisher, Version 4.0) with Affymetrix GeneChip Human Clariom™ S Assay. The gene expression changes were identified by pairwise comparison analyses (≥1.5-fold threshold), and expression patterns were analyzed by hierarchical clustering.

### shRNA lentiviral production

Lentiviral particles were produced in 293T cells using standard procedure with Lipofectamine 3000 (Invitrogen). In brief, 293T cells were transfected with 4.5 μg of packaging plasmid, pCMV-dR8.91, 0.5 μg of envelope plasmid, VSV-G/pMD2.G, and 1 μg of pLKO.1 viral plasmid of interest. Viral supernatant was harvested after 48 h of transfection and concentrated by Lenti-X Concentrator (TakaraBio) according to the manufacturer’s instructions.

### TEAD4 CRISPR/Cas9 KO stable clone

For CRISPR/Cas9 KO experiment, all-in-one CRISPR/Cas9 expression system (pAll-Cas9.pPuro) from Addgene with two TEAD4-specific sgRNAs (hTEAD4-sg1: 5′-ACTCATGGGACGTTCCGGCTTGG-3′ and hTEAD4-sg2: 5′-GGCCAGGACCTACCGTGTCCGGG-3′) were directly transfected in CWR22Rv1 cells with Lipofectamine 3000 (Invitrogen), which resulted in large deletion from Exon7 to Exon9 and followed by antibiotics selection with 2 μg/ml puromycin. After selection, single clone was selected by serial dilution in 96-well platform. The genomic DNA was isolated from each stable clones and deletion efficiency was evaluated by PCR with annealing temperature 62 °C (hTEAD4-gFwd: 5′-TGGCCTGTTAGCATTCACTAGCTAGGCGAG-3′, hTEAD4-gRev: 5′-CAGAGCAACCTTGGACTAAGGGTCGGTTG-3′) (data are shown in Supplementary Fig. 3d, right panel).

### Quantitative real-time PCR

Total RNA was extracted using the RNAspin Mini Kit (GE Healthcare) and concentration was measured by a Nanodrop 2000 instrument. One microgram of total RNA was for reverse transcription using iScript RT Supermix (Bio-Rad). Gene expression measured by qPCR data was collected by ViiA™ 7 Real-Time PCR System or Bio-Rad CFX Connect Real-time System with SYBR green PCR master mix (Bio-Rad) and confirmed in at least two independent experiments. Primer sequences are listed in Supplementary Tables 1–3.

### Chromatin immunoprecipitation

After 24-h arginine stimulation, samples were harvested and processed by ChIP with anti-acetyl-histone H3 antibody (Millipore 06-599) and rabbit IgG as isotype control as described by the manufacturer (Millipore Magna ChIP Kit #17-10085). After ChIP reaction, 1–10 ng of fragment DNA were used for ChIP-seq library construction and high-throughput DNA sequencing by using Illumina HiSeq2000 with 50 bp single-end sequencing (20 million reads per sample) at Phalanx Biotech Group.

### Seahorse assay

To measure cellular bioenergetics using extracellular flux, a Seahorse XF24 Extracellular Flux Analyzer (Agilent) was used. Cells were plated in XF 24-well cell culture microplates at a cell density of 5 × 10^4^ cells per well (Seahorse Bioscience) and incubated overnight. Next day, cells was replaced with unbuffered RPMI-1640 assay medium supplemented for 1-h equilibration. The OCR was measured over time at 6-min intervals. The first three measurements were conducted to establish a baseline rate, followed by three measurements after the addition of 2 μM oligomycin, to determine ATP turnover and the degree of proton leakage. Next, the maximal respiratory capacity was measured after the addition of the electron transport chain decoupler (FCCP, 0.5 μM). Finally, we administered 5 μM antimycin/rotenone to inhibit the flux of electrons through complex III and prevent oxygen consumption by the cytochrome *c* oxidase in the mitochondria. The value of OCR was determined by plotting the oxygen tension of the medium in the chamber as a function of time and normalized to total cell number. Absolute values of OCR were expressed as pmol per minute. The data were analyzed by the Seahorse Wave Desktop Software (version 2.4).

### MitoROS measurement

To evaluate the level of mitoROS, the mitochondrion-selective probes for total mitochondrial mass (MitoTracker Deep Red FM, Invitrogen) and ROS sensor by CellROX Oxidative stress reagent Probes (CellROX green reagent, Invitrogen C10444) were added to the media and incubated for 30 min at 37 °C. After incubation, cell nucleus was labeled with Hoechst dye and subjected to FACS analyses by NucleoCounter NC-3000^TM^ advanced image cytometer. Additionally, mitoROS was labeled with mitoROS dye (MitoSOX Red Mitochondrial superoxide indicator, Thermofisher) and visualized by Leica DMI6000 B inverted microscope with MetaMorph imaging series 7.8.

### ELISA assay

The total level of acetyl-CoA and histone H3 acetylation were determined by ELISA assay kits according to Abcam manufacturer’s protocol (ab87546 and ab8115102, respectively). The individual complex activities were analyzed by ELISA assay kits according to Abcam manufacturer’s protocol (complex I: ab108721, complex II: ab109908, complex IV: ab109909 and complex V: ab109714)

### Animal study

Six-week-old BACL/cAnN.Cg-Foxnlnu/CrlNarl male mice were purchased from National Laboratory Animal Center in Taiwan and housed in Animal Center at National Health Research Institutes under standard condition of the room temperature range between 20 and 25 °C, the relative ambient humidity of 50–70%, and semi-natural light cycle of 12:12-h light:dark. A half million of CWR22Rv1 PCa cells with luciferase gene^40^ were suspended in 100 µl of Matrigel™ (BD) and subcutaneously injected into mice. Tumor size was measured twice a week until a primary tumor size reached 20 mm in diameter. Tumor volume was calculated with the equation (*L* × *W*^2^)/2, where *L* is length and *W* is width of the tumor. Four weeks after tumor inoculation, the mice were first injected with D-luciferin (150 mg/Kg in saline) and then imaged by IVIS Lumina II Imaging System (Caliper Life Science). All animal procedures were conducted in accordance with the Institutional guidelines with animal welfare standards. The animal study was approved by the Institutional Animal Care and Use Committee at National Research Health Institutes (approval number: NHRI-IACUC-106077).

### Statistics and reproducibility

Statistical analysis was performed using GraphPad Prism 9 (Version 9.0.2). Data with error bar are presented as mean value ± SEM. Student’s two-tailed *t* test was used to determine the *p* value. Differences were considered statistically significant when the *p* value was <0.05. Results in Figs. 2f, 4a, b, e, f, i, 5c, 6b, d, e, and 7e and Supplementary Figs. 2e, 3a–d, and 4c, d, e are representative data of three independent repeats. And there were similar results in three independent repeats.

### Reporting summary

Further information on research design is available in the Nature Research Reporting Summary linked to this article.

## Supplementary information

Supplementary Information

Reporting Summary

## Data Availability

The expression profiling microarray data and ChIP-seq have been deposited in public Gene Expression Omnibus (GEO) database under the accession codes GSE149427 and GSE148908. Source data are provided with this paper.

## Source data

Source Data
