# Supplementary material for: Arginine is an epigenetic regulator targeting TEAD4 to modulate OXPHOS in prostate cancer cells
Source: Nat Commun. 2021 Apr 23;12:2398. doi: 10.1038/s41467-021-22652-9 (PMC8065123; doi:10.1038/s41467-021-22652-9)
Supplement: Supplementary file 1 — Supplementary Information [file 41467_2021_22652_MOESM1_ESM.pdf]

SUPPLEMENTARY INFROMATION

Arginine is an epigenetic regulator targeting TEAD4 to modulate OXPHOS in prostate cancer cells

Chia-Lin Chen, Sheng-Chieh Hsu, Tan-Ya Chung, Cheng-Ying Chu, Hung-Jung Wang, Pei-Wen Hsiao, Shauh-Der Yeh, David K. Ann, Yun Yen, Hsing-Jien Kung

Supplementary Figure 1-4
Supplementary Table 1-4
Supplementary reference

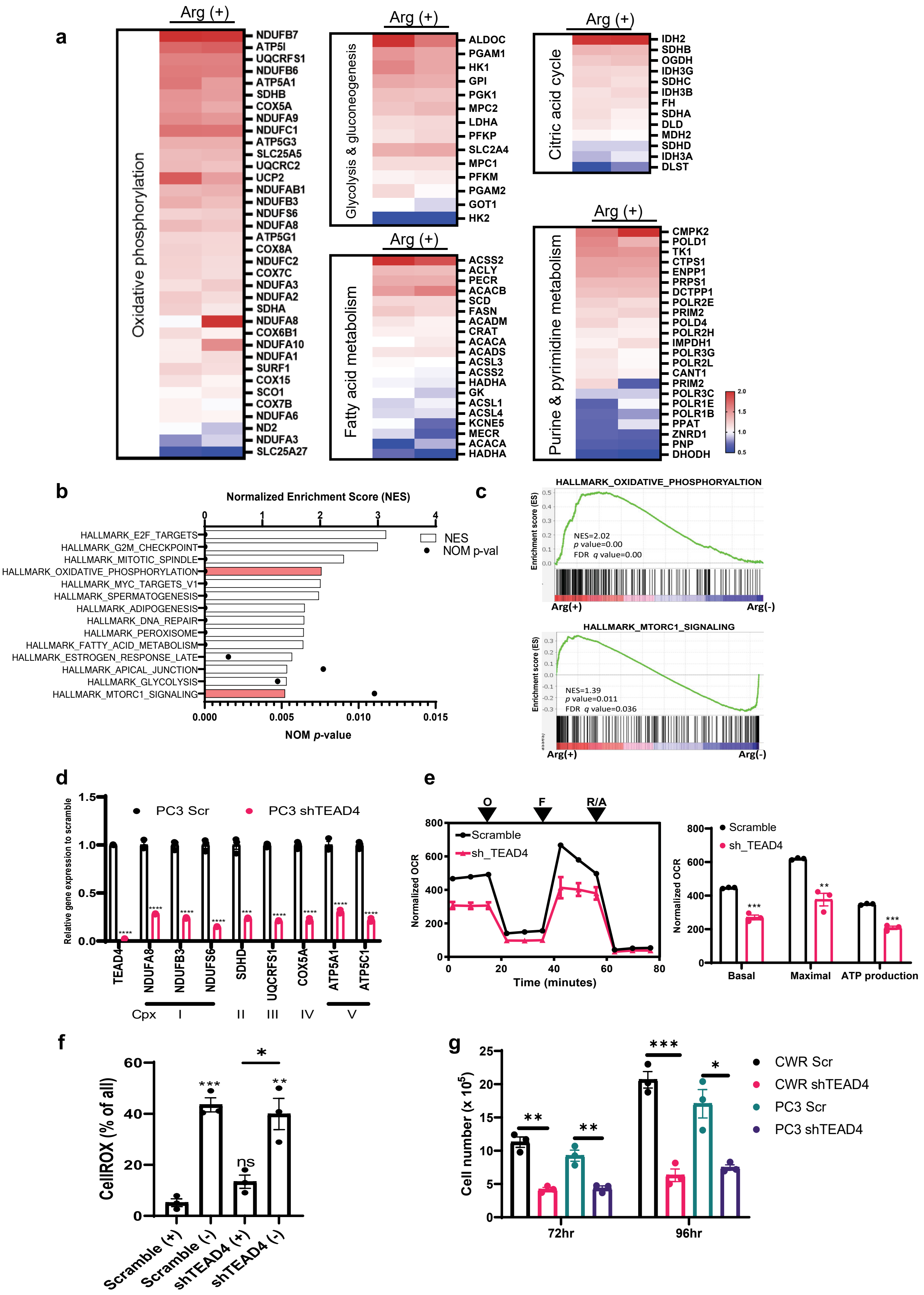

a
Arg (+)
Oxidative phosphorylation
NDUFB7
ATP5I
UQCRFS1
NDUFB6
ATP5A1
SDHB
COX5A
NDUFA9
NDUFC1
ATP5G3
SLC25A5
UQCRC2
UCP2
NDUFAB1
NDUFB3
NDUFS6
NDUFA8
ATP5G1
COX8A
NDUFC2
COX7C
NDUFA3
NDUFA2
SDHA
NDUFA8
COX6B1
NDUFA10
NDUFA1
SURF1
COX15
SCO1
COX7B
NDUFA6
ND2
NDUFA3
SLC25A27
Glycolysis & gluconeogenesis
ALDOC
PGAM1
HK1
GPI
PGK1
MPC2
LDHA
PFKP
SLC2A4
MPC1
PFKM
PGAM2
GOT1
HK2
Fatty acid metabolism
ACSS2
ACLY
PECR
ACACB
SCD
FASN
ACADM
CRAT
ACACA
ACADS
ACSL3
ACSS2
HADHA
GK
ACSL1
ACSL4
KCNE5
MECR
ACACA
HADHA
Citric acid cycle
IDH2
SDHB
OGDH
IDH3G
SDHC
IDH3B
FH
SDHA
DLD
MDH2
SDHD
IDH3A
DLST
Purine & pyrimidine metabolism
CMPK2
POLD1
TK1
CTPS1
ENPP1
PRPS1
DCTPP1
POLR2E
PRIM2
POLD4
POLR2H
IMPDH1
POLR3G
POLR2L
CANT1
PRIM2
POLR3C
POLR1E
POLR1B
PPAT
ZNRD1
PNP
DHODH
2.0
1.5
1.0
0.5
b
Normalized Enrichment Score (NES)
0
1
2
3
4
NES
NOM p-val
HALLMARK_E2F_TARGETS
HALLMARK_G2M_CHECKPOINT
HALLMARK_MITOTIC_SPINDLE
HALLMARK_OXIDATIVE_PHOSPHORYLATION
HALLMARK_MYC_TARGETS_V1
HALLMARK_SPERMATOGENESIS
HALLMARK_ADIPOGENESIS
HALLMARK_DNA_REPAIR
HALLMARK_PEROXISOME
HALLMARK_FATTY_ACID_METABOLISM
HALLMARK_ESTROGEN_RESPONSE_LATE
HALLMARK_APICAL_JUNCTION
HALLMARK_GLYCOLYSIS
HALLMARK_MTORC1_SIGNALING
0.000
0.005
0.010
0.015
NOM p-value
c
HALLMARK_OXIDATIVE_PHOSPHORYLTION
Enrichment score (ES)
NES=2.02
p value=0.00
FDR q value=0.00
Arg(+)
Arg(-)
HALLMARK_MTORC1_SIGNALING
NES=1.39
p value=0.011
FDR q value=0.036
Arg(+)
Arg(-)
d
Relative gene expression to scramble
PC3 Scr
PC3 shTEAD4
TEAD4
NDUFA8
NDUFB3
NDUFS6
SDHD
UQCRFS1
COX5A
ATP5A1
ATP5C1
Cpx
I
II
III
IV
V
e
O
F
R/A
Scramble
sh_TEAD4
Normalized OCR
Time (minutes)
Basal
Maximal
ATP production
f
CellROX (% of all)
ns
Scramble (+)
Scramble (-)
shTEAD4 (+)
shTEAD4 (-)
g
Cell number (x 10^5)
CWR Scr
CWR shTEAD4
PC3 Scr
PC3 shTEAD4
72hr
96hr

Supplementary Figure 1 Arginine epigenetically modulates mitochondrial OXPHOS genes (a) Heat map of Affymertrix microarray analysis shows arginine (Arg+) globally induced metabolic pathways, including oxidative phosphorylation pathway, glucose, fatty acid and DNA metabolic pathways in PC3 prostate cancer cells. The color key indicates the fold change to control. (b) Gene set enrichment assay (GSEA) of PC3 microarray data was performed using hallmark gene set collection. Normalized enrichment scores (NES) for pathways indicates the significantly difference in arginine stimulation group. (c) GSEA shows the genes associated with oxidative phosphorylation (upper panel) and mTORC1 pathway (lower panel) are enriched in arginine stimulation Arg (+) group. (d) Real-time PCR analysis of OXPHOS genes expression after silencing of TEAD4 *via* shRNA in PC3 cells. (e) Seahorse assay for mitochondrial respiration activities after silencing TEAD4 *via* shRNA in PC3 cells. O: oligomycin, F: FCCP, R/A: rotenone /antimycin. (f) Advance image cytometer analysis shows silencing of TEAD4 increased cellular ROS production in PC3 cells. (g) Cell number after silencing of TEAD4 by shRNA in CWR22Rv1 and PC3 cells at different time points in the presence of arginine. Data are presented as mean values + SEM of independent experiments (n=3 in d-g). *$p < 0.05$, **$p < 0.01$, ***$p < 0.001$, ****$p < 0.0001$, ns: not significant, using unpaired two-tailed Student's t test. Source data are provided as a Source Data file.

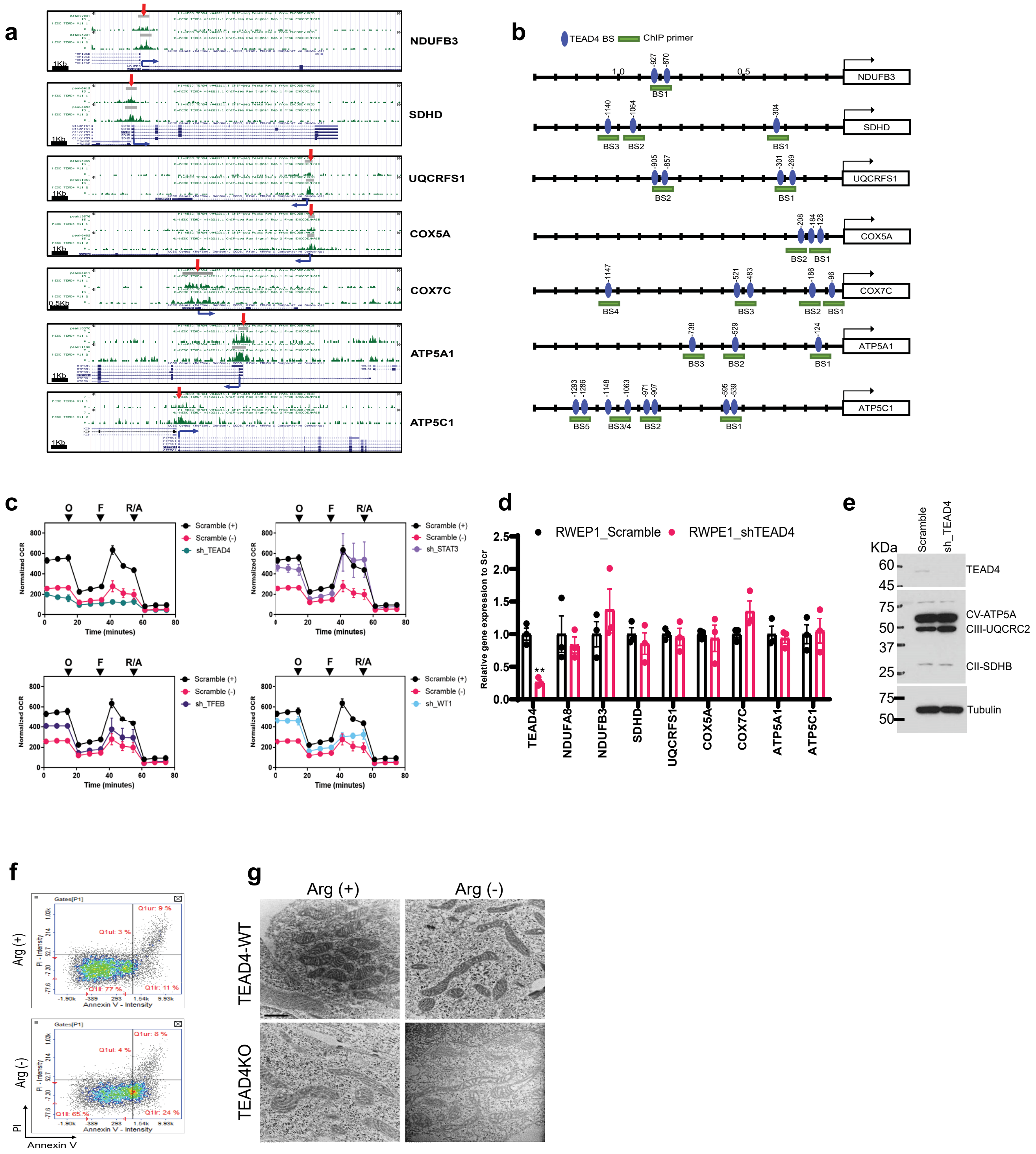
a
NDUFB3
SDHD
UQCRFS1
COX5A
COX7C
ATP5A1
ATP5C1
1Kb
0.5Kb
b
TEAD4 BS
ChIP primer
NDUFB3
SDHD
UQCRFS1
COX5A
COX7C
ATP5A1
ATP5C1
BS1
BS2
BS3
BS4
BS5
BS3/4
c
O
F
R/A
Scramble (+)
Scramble (-)
sh_TEAD4
sh_STAT3
sh_TFEB
sh_WT1
Normalized OCR
Time (minutes)
d
RWEP1_Scramble
RWPE1_shTEAD4
Relative gene expression to Scr
TEAD4
NDUFA8
NDUFB3
SDHD
UQCRFS1
COX5A
COX7C
ATP5A1
ATP5C1
e
KDa
Scramble
sh_TEAD4
TEAD4
CV-ATP5A
CIII-UQCRC2
CII-SDHB
Tubulin
f
Arg (+)
Arg (-)
PI
Annexin V
g
Arg (+)
Arg (-)
TEAD4-WT
TEAD4KO

Supplementary Figure 2 Arginine targets TEAD4 to modulate the mitochondrial functions (a) TEAD4 ChIP-seq distribution at representative OXPHOS genes loci from ENCODE database (ENCFF000OWA and ENCFF000OWD), visualized by UCSC Genome Browser (https://genome.ucsc.edu). (b) Predication of TEAD4 binding sites (BS in blue) on OXPHOS promoter regions by FIMO program. The green box indicates the target sites of ChIP-qPCR. (c) Individual shRNA plot for seahorse assay in Figure 3c. (d) TEAD4 was knock-down in non-arginine auxotrophic epithelial prostate cells, RWPE1, *via* lentivirus shRNA infection for 24 hours, following antibiotics selection for one week. OXPHOS genes expression was determined by quantitative real-time PCR and the protein level was evaluated by immunoblot (e). (f) Apoptosis analysis by double staining of annexin V and propidium iodide (PI) staining after arginine deprivation for 48-hours. (g) Ultrastructure of mitochondrial morphology after 48-hours arginine deprivation in wild type (WT) or TEAD4 knock-out (TEAD4KO) cells (scale bar=500nm). Data are presented as mean values + SEM of independent experiments (n=3 in d). ***p* < 0.01, using unpaired two-tailed Student's t test. Source data are provided as a Source Data file.

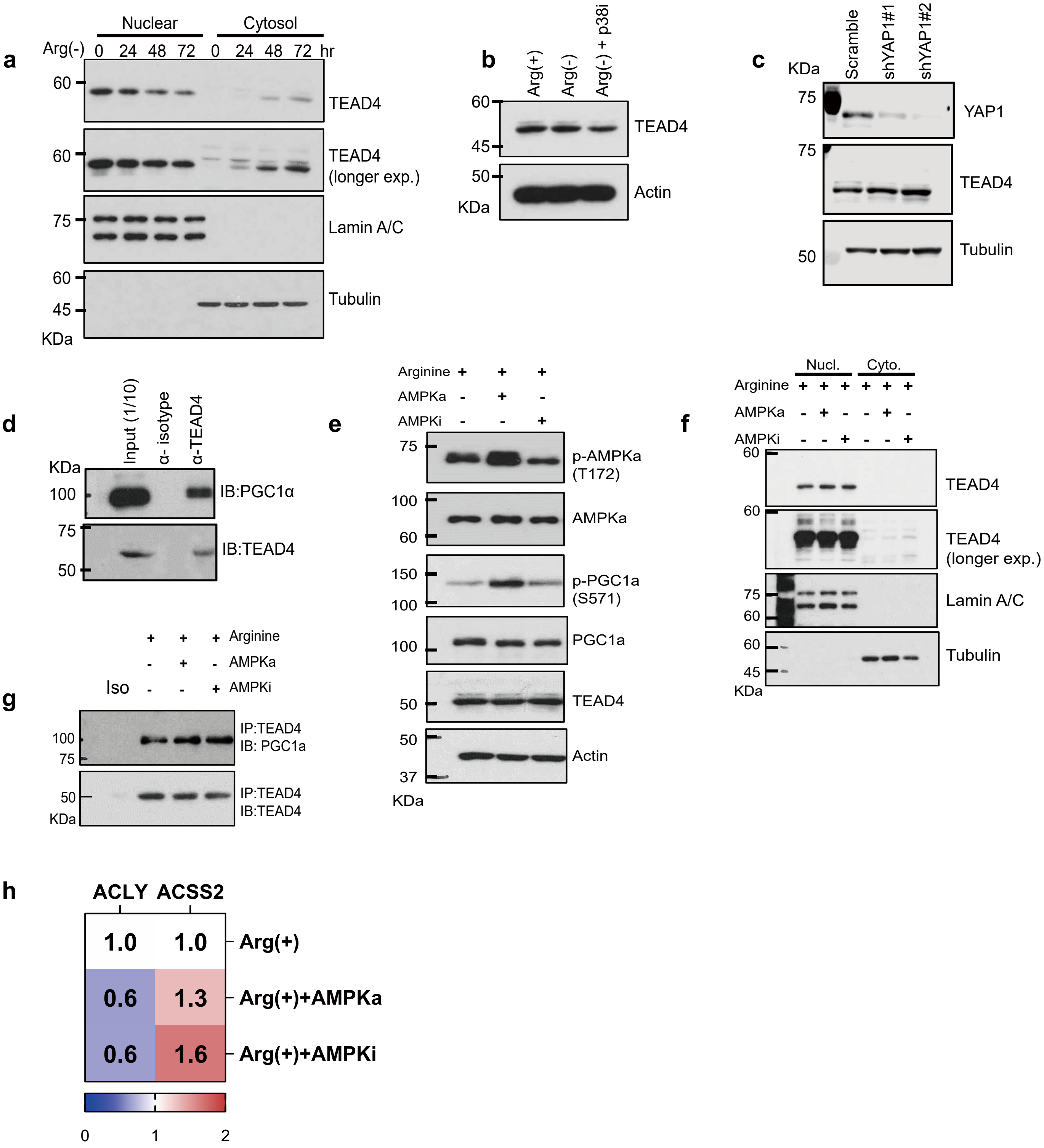

a
Nuclear
Cytosol
Arg(-) 0 24 48 72 0 24 48 72 hr
60
TEAD4
60
TEAD4 (longer exp.)
75
Lamin A/C
60
45
Tubulin
KDa
b
Arg(+)
Arg(-)
Arg(-) + p38i
60
45
TEAD4
50
Actin
KDa
c
KDa
Scramble
shYAP1#1
shYAP1#2
75
YAP1
75
TEAD4
50
Tubulin
d
Input (1/10)
α- isotype
α-TEAD4
KDa
100
IB:PGC1α
75
50
IB:TEAD4
e
Arginine + + +
AMPKa - + -
AMPKi - - +
75
p-AMPKa (T172)
100
60
AMPKa
150
100
p-PGC1a (S571)
100
PGC1a
50
TEAD4
50
37
Actin
KDa
f
Nucl.
Cyto.
Arginine + + + + + +
AMPKa - + - - + -
AMPKi - - + - - +
60
TEAD4
60
TEAD4 (longer exp.)
75
60
Lamin A/C
60
45
Tubulin
KDa
g
+ + + Arginine
- + - AMPKa
Iso - - + AMPKi
100
75
IP:TEAD4 IB: PGC1a
50
KDa
IP:TEAD4 IB:TEAD4
h
ACLY ACSS2
1.0 1.0 Arg(+)
0.6 1.3 Arg(+)+AMPKa
0.6 1.6 Arg(+)+AMPKi
0 1 2

Supplementary Figure 3 Arginine mediated nuclear retention of TEAD4 (a) Immunoblotting shows TEAD4 location after arginine deprivation at different time pointed (related to figure 4e). (b) Immunoblotting of total TEAD4 expression after treatment (related to figure 4f). (c) YAP1 was knocked down by two individual YAP1 shRNA clones via lentivirus transduction for 24 hours, following antibiotics selection for one week. Total TEAD4 protein expression was evaluated by immunoblotting. (d) The interaction of TEAD4 with PGC1a was evaluated by immunoprecipitation. Total lysate was first incubated with isotype or TEAD4 antibody overnight and then pull-down by magnetic beads. Interaction was evaluated by PGC1a antibody. 10% of total protein as internal control. (e) In the presence of arginine, cells were treated with either AMPK activator, Metformin (10mM), or AMPK inhibitor, Dorsomorphin (10μM), for 24hours. The downstream target, phosphor-PGC1a (S571) was confirmed by immunoblot. However, the total level of TEAD4 was not affected. (f) The immunoblot of cell fractionation shows AMPK did not affection TEAD4 translocation in the presence of arginine. (g) The immunoprecipitation shows AMPK did not affect the interaction of TEAD4 with PGC1a in the presence of arginine. (h) The quantitative real-time PCR shows AMPK did not correlate with the expression of ACLY or ACSS2 in the presence of arginine. The color key indicates the relative gene expression to control. Source data are provided as a Source Data file.

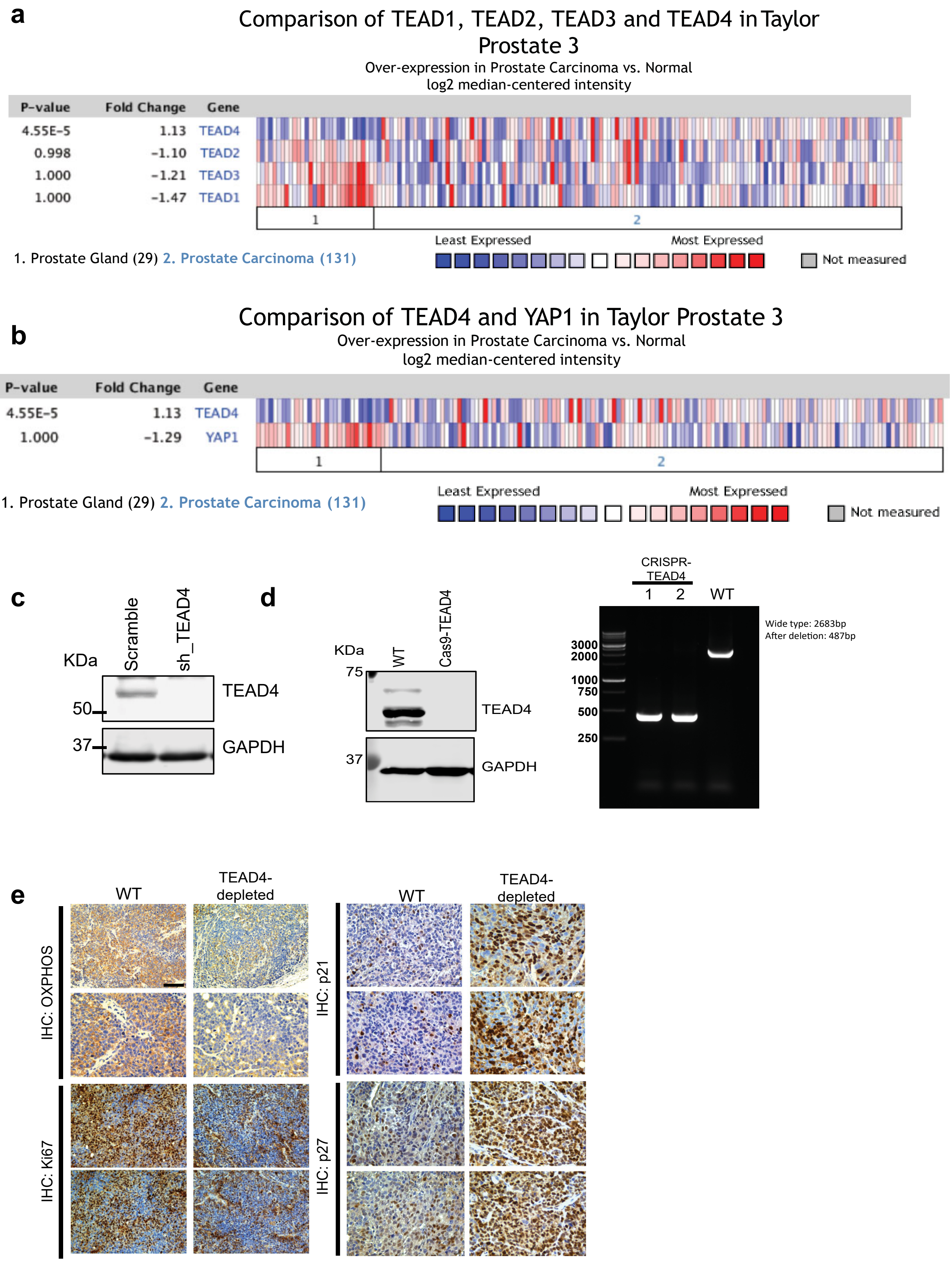

a
Comparison of TEAD1, TEAD2, TEAD3 and TEAD4 in Taylor Prostate 3
Over-expression in Prostate Carcinoma vs. Normal
log2 median-centered intensity
P-value
Fold Change
Gene
4.55E-5
1.13
TEAD4
0.998
-1.10
TEAD2
1.000
-1.21
TEAD3
1.000
-1.47
TEAD1
1
2
Least Expressed
Most Expressed
Not measured
1. Prostate Gland (29) 2. Prostate Carcinoma (131)
b
Comparison of TEAD4 and YAP1 in Taylor Prostate 3
Over-expression in Prostate Carcinoma vs. Normal
log2 median-centered intensity
P-value
Fold Change
Gene
4.55E-5
1.13
TEAD4
1.000
-1.29
YAP1
1
2
1. Prostate Gland (29) 2. Prostate Carcinoma (131)
Least Expressed
Most Expressed
Not measured
c
KDa
Scramble
sh_TEAD4
50
37
TEAD4
GAPDH
d
KDa
WT
Cas9-TEAD4
75
37
TEAD4
GAPDH
CRISPR-TEAD4
1
2
WT
3000
2000
1000
750
500
250
Wide type: 2683bp
After deletion: 487bp
e
WT
TEAD4-depleted
IHC: OXPHOS
IHC: Ki67
IHC: p21
IHC: p27

Supplementary Figure 4 TEAD4 could be a potential target for cancer therapy (a) Oncomine database (www.oncomine.org) shows TEADs expression in Taylor Prostate cancer library. (b) Oncomine database shows TEAD4 and YAP1 expression in Taylor prostate cancer library. (c) Western blotting shows the knock-down efficiency of shRNA_TEAD4 CWR22Rv1 cells before subcutaneous injection. (d) Knock-out efficiency of CRISPR-Cas9 TEAD4 (left panel) CWR22Rv1 cells before subcutaneous injection as well as PCR confirmation (right panel). (e) IHC staining of pan-OXPHOS protein, CDK inhibitors, p21 and p27, and proliferation marker, Ki67 in mouse tumor samples (scale bar=50μm). Source data are provided as a Source Data file.

Supplementary Table 1 Target OXPHOS genes in TEAD4 ChIP-seq datasets from the ENCODE dataset

Complex	I		II	III	IV	V
Targets	NDUFA1<br>NDUFA2<br>NDUFA3<br>NDUFA4<br>NDUFA4L2<br>NDUFA5<br>NDUFA6<br>NDUFA7<br>NDUFA8<br>NDUFA9<br>NDUFA10<br>NDUFA11<br>NDUFA12<br>NDUFA13<br>NDUFAB1<br>NDUFAF1<br>NDUFAF2<br>NDUFAF3<br>NDUFAF4<br>NDUFAF5<br>NDUFAF6<br>NDUFAF7	NDUFB1<br>NDUFB2<br>NDUFB3<br>NDUFB5<br>NDUFB6<br>NDUFB7<br>NDUFB9<br>NDUFB10<br>NDUFB11<br>NDUFC1<br>NDUFC2<br>NDUFC2-KCTD14<br>NDUFS1<br>NDUFS3<br>NDUFS4<br>NDUFS5<br>NDUFS6<br>NDUFS7<br>NDUFS8<br>NDUFV1<br>NDUFV2<br>NDUFV3	SDHA<br>SDHAF1<br>SDHAF2<br>SDHAF3<br>SDHAF4<br>SDHAP1<br>SDHAP3<br>SDHB<br>SDHC<br>SDHD	UQCR10<br>UQCR11<br>UQCRB<br>UQCRC1<br>UQCRC2<br>UQCRFS1<br>UQCRH<br>UQCRHL<br>UQCRQ<br>CYC1	COX10<br>COX11<br>COX14<br>COX15<br>COX16<br>COX17<br>COX18<br>COX19<br>COX20<br>COX4I1<br>COX5A<br>COX5B<br>COX6A1<br>COX6B1<br>COX6C<br>COX7A2<br>COX7A2L<br>COX7B<br>COX7C<br>COX8A	ATP5B<br>ATP5C1<br>ATP5D<br>ATP5E<br>ATP5O<br>ATP5F1<br>ATP5G1<br>ATP5G2<br>ATP5G3<br>ATP5H<br>ATP5I<br>ATP5J<br>ATP5J2<br>ATP5J2-PTCD1<br>ATP5L<br>ATP5L2<br>ATP5S<br>ATP5SL

Supplementary Table 2 Primer list For ChIP-histone H3 acetylation on OXPHOS promoter region

Promoter region	Forward primer sequence (5' to 3')	Reverse primer sequence (5' to 3')
NDUFB3-pro	GAT GCT ACC TGG TTT ACG AGA G	GGA GGC AAA GTG CTA GAT GAA
NDUFS6-pro	GAA CCA CTG GGT ACT CCT AAT C	TGC AGC CTC ATG CAG TT
SDHD-pro	CTA ACT AGT CTC CCG TAC CCT T	GCT CAG GTG CCA GGT ATA TAA G
UQCRFS1-pro	CAA GAT GTG AAG CAG GGT AAT C	GAG CTC TGT GCA TCC TCT AA
COX5A-pro	TGT GGC TCA TGC CTG TAAT C	ACC ACG CCT GGC TAA TTT
COX7C-pro	CCC TTC TAG TGC TCC TTG ATA C	TTC CCA ACA TGG CTG AGT T
ATP5A1-pro	ACC ACA ACT CCC AGA AAT CC	CCG CCA CTT TAC TAG GAA CTC
ATP5C1-pro	GGT ACA GTG TAG GTG CTC AAT AA	AGA AAC AGG GTG GTT AGG ATT C

Supplementary Table 3 Primer list for ChIP-TEAD4 on OXPHOS promoter region

TEAD4 binding site	Forward primer sequence (5' to 3')	Reverse primer sequence (5' to 3')
NDUFB3_TEAD4_BS1	GGA GGA AGG GAT CTA CCA ACT A	GGG TCA AGG CTG ACA ACA T
SDHD_TEAD4_BS1	GGA CAG GAG AGC CAT AAC TTT G	ACC TTC CTT GGT GGT CCT
SDHD_TEAD4_BS2	TGA ACC AAC TAG GGA ACC AAT TA	ATG GAA GAA TTA CCT TGT CTA GGG
SDHD_TEAD4_BS3	AGA TCC TCG TCC CTA CAG ATT	TTG GTT CCC TAG TTG GTT CAC
UQCRFS1_TEAD4_BS1	GCGAT TACTA TGTGC TGGAC T	GGCCG ACTTT ACGAT CCTTT A
UQCRFS1_TEAD4_BS2	ACT CTA GAC TCA GTT CTC TGC T	TTG GCC TCA GTT TGA TCC TAA T
COX5A_TEAD4_BS1	ACA AGA TCA CGG TCG GTT G	CCT TGT CTC TGG TCA GGT G
COX5A_TEAD4_BS2	GCT GGG ATT ACA GGC ATG A	CCC AGG AGG GCA CTT AAT C
COX7C_TEAD4_BS1	CGA AAC TAC ATT TCC CAC AAT CC	CTT GAG GAA ACG CGA CAA AG
COX7C_TEAD4_BS2	AGG GAT GGG TTT ATT GTC CTT T	CCC GAA GGA TTG TGG GAA AT
COX7C_TEAD4_BS3	TTC CCT GCC CTT TGT TCT T	GCA CAA AGG ACT TCA GAG AGA
COX7C_TEAD4_BS4	GGC ATG GTT GTT CTC TTC CA	ATT GCC TCA CGT GTC TCA TTT A
ATP5A1_TEAD4_BS1	CCT GGG AAC TGC TGC TTA G	TGG TCC TGG CAT TCG TTA TAC
ATP5A1_TEAD4_BS2	AGC TGG GAA TCT TTA ACC TGA G	TCA GAC CCT CTG AGT CGA AA
ATP5A1_TEAD4_BS3	ATG ACT CTT CTA GGC CTC CTT	ACC TAG GTG ATG GGA TGA TAG T
ATP5C1_TEAD4_BS1	CCG GGC GTC TAG TAT TGC	CAG CCT GTG TTC TGC GA
ATP5C1_TEAD4_BS2	AGA CTC CTT GAG CCA CTC TAT	CTT ACC AAC CGT GTG GTC TT
ATP5C1_TEAD4_BS3	GCA TTC AAT CAA CCA CCT CTT T	TAG AGT GGC TCA AGG AGT CT
ATP5C1_TEAD4_BS4	CTT CCT TCA CTT GCA GAA ACA C	GTG CAG GCT AGT GAT GTG AT
ATP5C1_TEAD4_BS5	CAC CCA GTC TAC TGC ACT TTA T	TGC AAG TGA AGG AAG AGG TAT T

Supplementary Table 4 Primer list for qPCR assay

Genes	Forward primer sequence (5' to 3')	Reverse primer sequence (5' to 3')
NDUFA8	CCT TTA CCG GAG AAT CCC TAT C	CCA CGG ACC CAT CTT TAC TT
NDUFAB1	TAT AGC GAC ATG CCT CCT TTG	GAA AGC TTC TCT GGG TCA ATC T
NDUFB3	GGA CAT GAG CAT GGA CAT CAT A	GCC AGC TTC TTC TGG ATA GTT
NDUFS6	CAC ACT GGC CAG GTT TAT GA	GGG CTG CTC TGC TAT CAA AT
SDHD	CGA GAG GGT TGT CAG TGT TT	CCA GTG ACC ATG AAG AGT GAG
UQCRFS1	CCG ATA TTC CAG AAG GCA AGA A	TGG GTC CCT CAA CTG TGA TA
COX5A	GCT CGC TGG GTA ACA TAC TT	GGG CTC TGG AAC CAT ATC ATA G
COX7C	GTC CGT AGG AGC CAC TAT GA	GTG TAG CAA ATG CAG ATC CAA AG
ATP5A1	GAG GAC TGT GTG GTG CTA TTC	CTT TCC CAG CTG CTG TTA GT
ATP5C1	GAG GAC TGT GTG GTG CTA TTC	CTT TCC CAG CTG CTG TTA GT
ACSS1	GGT ACT GGG AGA CAG TAG AGA G	CCC AGG CAT CAC CGT ATT T
ACSS2	GCT GCA GTC TTC TCA TCA CTA C	GAA ACC CTT CTC CTG ACA CTT C
ACLY	AAC ATC CTG GCC TCC AAA C	CGT GGT CCG AGA GAT GAT ATT G
HAT1	TAT TGC TGG TAG CCT GTC AAC	CCT CAA CAT CAT CTG CCT CTA C
KAT2A	GGG TTT CTC CAA GGA CAT CAA	GAT TCA GCT CAC ACT CCA TCA G
KAT2B	GCC GTG TTA TTG GTG GTA TCT	TAG CCC TTG ACT TGC TCA TTT
KAT3A	GGA CCC AGA GTC ATT ACC TTT C	GAG AGG TCC ATG GGA TTC TTT AC
KAT3B	GCA GCA ACA GGT GCT TAG TA	CAG GGA TGG GTT GTG GAT TAG
KAT5	TCC TCC AGG CAA TGA GAT TTA C	AGA CAC AGG TTC TGG GAA TAA C
KAT6A	ATG GAT GTG CCT TCC GTA TC	TCG TAA CTG CTT GGG TTC TC
KAT6B	AAA GGG ACA CCC GAG TTA TG	CTA GAG GAG GAG GAG TGA GAA A
KAT7	CTG ACC CAA GGA GTT CTG TTA TG	AGC ACT AGT ACC ACG CTT AGA
KAT8A	CGA TCA CCA AGG TGA AGT ATG T	CTT GGG CTG TTT CCC ATA GT
KAT9	CCA AAC GTG GGA CTA GAA AGA G	CGA ATC ACC AGG GTA GGA TAG A
TEAD1	CTG AGT CGC AGT TAC CAC CA	AGC CTG GAG CCT TTT CAA G
TEAD2	ACA TGA TGA ACA GCG TCC TG	CAG CAG TTC CTG GGT GTC TC
TEAD3	CAT CGA GCA GAG CTT CCA G	CGT GCA ATC AAC TCA TTT CG
TEAD4	GCC TTC CAC AGT AGC ATG G	AAA GCT CCT TGC CAA AAC C
YAP1	GCA AAT TCT CCA AAA TGT CAG G	CGG GAG AAG ACA CTG GAT TT

Supplementary references for Bioinformatic packages

ChIP-seq analysis[1-8] and Motif analysis[9-11]

1. Bolger, A.M., Lohse, M. & Usadel, B. Trimmomatic: a flexible trimmer for Illumina sequence data. Bioinformatics 30, 2114-2120 (2014).
2. Langmead, B., Trapnell, C., Pop, M. & Salzberg, S.L. Ultrafast and memory-efficient alignment of short DNA sequences to the human genome. Genome Biol 10, R25 (2009).
3. Langmead, B. & Salzberg, S.L. Fast gapped-read alignment with Bowtie 2. Nat Methods 9, 357-359 (2012).
4. Zhang, Y. et al. Model-based analysis of ChIP-Seq (MACS). Genome Biol 9, R137 (2008).
5. Feng, J., Liu, T., Qin, B., Zhang, Y. & Liu, X.S. Identifying ChIP-seq enrichment using MACS. Nat Protoc 7, 1728-1740 (2012).
6. Ross-Innes, C.S. et al. Differential oestrogen receptor binding is associated with clinical outcome in breast cancer. Nature 481, 389-393 (2012).
7. Yu, G., Wang, L.G. & He, Q.Y. ChIPseeker: an R/Bioconductor package for ChIP peak annotation, comparison and visualization. Bioinformatics 31, 2382-2383 (2015).
8. Ramirez, F. et al. deepTools2: a next generation web server for deep-sequencing data analysis. Nucleic Acids Res 44, W160-165 (2016).
9. Bailey, T.L. DREME: motif discovery in transcription factor ChIP-seq data. Bioinformatics 27, 1653-1659 (2011).
10. Grant, C.E., Bailey, T.L. & Noble, W.S. FIMO: scanning for occurrences of a given motif. Bioinformatics 27, 1017-1018 (2011).
11. Machanick, P. & Bailey, T.L. MEME-ChIP: motif analysis of large DNA datasets. Bioinformatics 27, 1696-1697 (2011).

Ingenuity of pathway analysis (IPA)[12], Oncomine[13], ENCODE[14, 15] and GSEA[16]

12. Kramer, A., Green, J., Pollard, J., Jr. & Tugendreich, S. Causal analysis approaches in Ingenuity Pathway Analysis. Bioinformatics 30, 523-530 (2014).
13. Rhodes, D.R. et al. ONCOMINE: a cancer microarray database and integrated data-mining platform. Neoplasia 6, 1-6 (2004).
14. Consortium, E.P. An integrated encyclopedia of DNA elements in the human genome. Nature 489, 57-74 (2012).
15. Davis, C.A. et al. The Encyclopedia of DNA elements (ENCODE): data portal update. Nucleic Acids Res 46, D794-D801 (2018).

16. Subramanian, A. et al. Gene set enrichment analysis: a knowledge-based approach for interpreting genome-wide expression profiles. Proc Natl Acad Sci U S A 102, 15545-15550 (2005).
