# Supplementary material for: Arginine is an epigenetic regulator targeting TEAD4 to modulate OXPHOS in prostate cancer cells
Source: Nat Commun. 2021 Apr 23;12:2398. doi: 10.1038/s41467-021-22652-9 (PMC8065123; doi:10.1038/s41467-021-22652-9)
Supplement: Supplementary file 2 — Reporting Summary [file 41467_2021_22652_MOESM2_ESM.pdf]

nature research

Corresponding author(s): Chia-Lin Chen, Hsing-Jien Kung

Last updated by author(s): Mar 8, 2021

Reporting Summary

Nature Research wishes to improve the reproducibility of the work that we publish. This form provides structure for consistency and transparency in reporting. For further information on Nature Research policies, see our Editorial Policies and the Editorial Policy Checklist.

Statistics

For all statistical analyses, confirm that the following items are present in the figure legend, table legend, main text, or Methods section.

n/a	Confirmed	
☐	☒	The exact sample size (n) for each experimental group/condition, given as a discrete number and unit of measurement
☐	☒	A statement on whether measurements were taken from distinct samples or whether the same sample was measured repeatedly
☐	☒	The statistical test(s) used AND whether they are one- or two-sided<br>*Only common tests should be described solely by name; describe more complex techniques in the Methods section.*
☒	☐	A description of all covariates tested
☐	☒	A description of any assumptions or corrections, such as tests of normality and adjustment for multiple comparisons
☐	☒	A full description of the statistical parameters including central tendency (e.g. means) or other basic estimates (e.g. regression coefficient) AND variation (e.g. standard deviation) or associated estimates of uncertainty (e.g. confidence intervals)
☐	☒	For null hypothesis testing, the test statistic (e.g. F, t, r) with confidence intervals, effect sizes, degrees of freedom and P value noted<br>*Give P values as exact values whenever suitable.*
☒	☐	For Bayesian analysis, information on the choice of priors and Markov chain Monte Carlo settings
☒	☐	For hierarchical and complex designs, identification of the appropriate level for tests and full reporting of outcomes
☒	☐	Estimates of effect sizes (e.g. Cohen's d, Pearson's r), indicating how they were calculated

Our web collection on statistics for biologists contains articles on many of the points above.

Software and code

Policy information about availability of computer code

Data collection	qPCR data was collected by ViiA™ 7 Real-Time PCR System or Bio-Rad CFX Connect Real-time System. Seahorse data was collected by Agilent Seahorse XFe24 analyzer with Seahorse Wave Desktop Software(version 2.4). Western blot images were scanned by LI-COR Odyssey Infrared Imaging System. Cell number and the level of immunofluorescent dye were collected by NucleoCounter® NC-3000™ Advanced Image Cytometer. Immunofluorescent images were collected by Leica DMI6000 B inverted microscope with MetaMorph imaging series 7.8.
Data analysis	GraphPad Prim 9 (Version 9.0.2), Transcriptiome Analysis Console (Version 4.0). Ingenuity Pathway analysis (IPA). Gene set enrichment analysis (GSEA Version 4.1.0)

For manuscripts utilizing custom algorithms or software that are central to the research but not yet described in published literature, software must be made available to editors and reviewers. We strongly encourage code deposition in a community repository (e.g. GitHub). See the Nature Research guidelines for submitting code & software for further information.

Data

Policy information about availability of data

All manuscripts must include a data availability statement. This statement should provide the following information, where applicable:

- Accession codes, unique identifiers, or web links for publicly available datasets
- A list of figures that have associated raw data
- A description of any restrictions on data availability

The expression profiling microarray data and ChIP-seq have been deposited in public Gene Expression Omnibus (GEO) database under the accession code GSE149427 and GSE148908. The microarray and CHIP-seq data referenced during the study are available in a public repository from the website (https://www.ncbi.nlm.nih.gov/geo/query/acc.cgi?acc=GSE149427 and https://www.ncbi.nlm.nih.gov/geo/query/acc.cgi?acc=GSE148908).

The prostate cancer libraries, including Taylor Prostate 3 cancer library and Setlur prostate cancer library, are available from Oncomine cancer database (https://www.oncomine.org).

Field-specific reporting

Please select the one below that is the best fit for your research. If you are not sure, read the appropriate sections before making your selection.

☒ Life sciences ☐ Behavioural & social sciences ☐ Ecological, evolutionary & environmental sciences

For a reference copy of the document with all sections, see nature.com/documents/nr-reporting-summary-flat.pdf

Life sciences study design

All studies must disclose on these points even when the disclosure is negative.

Sample size	No statistical method was used to predetermine sample sizes. For in vitro experiments, we chose sample sizes based on our previous experience (PMID: 24692592) . For in vivo xenograft experiments, six biologically individual tumors were used to reach the statistical power(PMID: 29386977).
Data exclusions	No data were excluded from the analysis.
Replication	All in vitro experiments in this study have been done more than twice with triplicates. For in vivo xenograft experiments, six biologically individual tumors were used for statistical power. More detail was described in method section and figure legends as well.
Randomization	For in vitro study, cells were treated randomized. For in vivo study, animals injected with cancer cells were chosen randomly.
Blinding	No blinding test was used in this study since the same investigator for both experimental design and conducting the experiments.

Reporting for specific materials, systems and methods

We require information from authors about some types of materials, experimental systems and methods used in many studies. Here, indicate whether each material, system or method listed is relevant to your study. If you are not sure if a list item applies to your research, read the appropriate section before selecting a response.

Materials & experimental systems

n/a	Involved in the study	
☐	☒	Antibodies
☐	☒	Eukaryotic cell lines
☒	☐	Palaeontology and archaeology
☐	☒	Animals and other organisms
☒	☐	Human research participants
☒	☐	Clinical data
☒	☐	Dual use research of concern

Methods

n/a	Involved in the study	
☐	☒	ChIP-seq
☒	☐	Flow cytometry
☒	☐	MRI-based neuroimaging

Antibodies

Antibodies used	TEAD4(#ab58310), pan-OXPHOS (#ab110413) and Ki67(#ab15580) were purchased from Abcam. p-mTOR (T2446) (#09-345), Acetyl-Histone H3 (#06-599) were purchased from Millipore. Phosphor-PGC1a (#AF6650) was purchased form R&D. mTOR(#2938), p-mTOR(S2448)(#5536), YAP(#14074), p-YAP(S127)(#13008), p-YAP(S397)(#13619), p38(#8690), p-p38 (T180/182)(#4511), Acetyl-Histone H3 (Lys9)(#9649), Acetyl-Histone H3 (Lys14)(#7627), Acetyl-Histone H3 (Lys18)(#13998), Acetyl-Histone H3 (Lys27)(#8173), Acetyl-Histone H3 (Lys56)(#4243), Histone H3(#4499), Acetyl-Histone H2A (Lys5)(#2579), Acetyl-Histone H2B (Lys5)(#12799), Histone H2A(#12349), Histone H2B(#12364), Acetyl-Histone H4 (Lys5)(#8647), Acetyl-Histone H4 (Lys8)(#2594), Acetyl-Histone H4 (Lys12)(#13944), Acetyl-Histone H4(#2935), PGC1a(#2178), p21(#2974), p27(#83630) were purchased form Cell Signaling. All antibodies were used at a dilution of 1:1000 for immunoblotting and 1:250 for immunofluorescent staining.
Validation	All antibodies are commercially available and have been tested for species reactivity and validated by the manufacturers. The statements of validation of each primary antibody for the species and application are also available on the manufacturer's website.

Eukaryotic cell lines

Policy information about cell lines

Cell line source(s)	CWR22Rv1(ATCC CRL-250), PC3 (ATCC CRL-1435), LNCaP (ATCC CRL-1740), DU145 (ATCC HTB-81), C4-2B (ATCC CRL-3315), and immortalized prostate cell line, RWPE-1(ATCC CRL-11609), were purchased from ATCC.

Authentication	The cell lines are authenticated by manufacturer (ATCC).
Mycoplasma contamination	All cell lines are routinely checked the mycoplasma contamination by e-Myco Mycoplasma PCR detection kit twice a year in our lab to ensure the quality of cell lines, but not every time before the experiments. The mycoplasma-positive cell ine will be discard.
Commonly misidentified lines (See ICLAC register)	No commonly misidentified lines were used in this study.

Animals and other organisms

Policy information about studies involving animals; ARRIVE guidelines recommended for reporting animal research

Laboratory animals	6-week-old BACL/cAnN.Cg-Foxnlnu/CrlNarl male mice were purchased from National Laboratory Animal Center in Taiwan and housed in Animal Center at National Health Research Institutes under standard condition of the room temperature range between 20-25oC, the relative ambient humidity of 50-70% and semi-natural light cycle of 12:12 hours light:dark.
Wild animals	No wild animals were used in this study
Field-collected samples	No field-collected samples were used in this study
Ethics oversight	The animals involved in in vivo study were performed according to the approved IACUC protocol (NHRI-IACUC-106077) by Animal Care and Use Committee at National Research Health Institutes.

Note that full information on the approval of the study protocol must also be provided in the manuscript.

ChIP-seq

Data deposition

☒ Confirm that both raw and final processed data have been deposited in a public database such as GEO.

☒ Confirm that you have deposited or provided access to graph files (e.g. BED files) for the called peaks.

Data access links *May remain private before publication.*	https://www.ncbi.nlm.nih.gov/geo/query/acc.cgi?acc=GSE148908
Files in database submission	Six raw ChIP-seq (total acetylated H3 antibody, isotype control and input sample) and the differential analysis including annotation are available in GSE148908 dataset
Genome browser session (e.g. UCSC)	The analyzed Bedgraph files, except input samples, were deposited in https://drive.google.com/open?id=1xsREV4uvYARFPm2gMnOUoHrjVVxWeLTf and can be visualized by Integrative Gebomic Viewer (IGV) or UCSC genome browser.

Methodology

Replicates	Each group of samples were combined with two technical replicates
Sequencing depth	The ChIP-sequencing was using illumina HiSeq2500 as platform (50bp of single-end sequencing) for total reads 20 millions reads per samples. Mapping ratio is between 92.91% ~ 96.16%.
Antibodies	Acetyl-Histone H3 (#06-599, ChIP-grade) was purchased from Millipore
Peak calling parameters	Total peak numbers were contained from the peak calling meta-data using MACS2 with default settings. Inputs were used as internal controls during peak calling step to assess background signals/peaks.
Data quality	There are 12679 peaks in control group and 14714 peaks in treated group at 5% FDR above 2-fold enrichment.
Software	The ChIP-seq data was first trimmed by Trimmomatic to remove adapter sequences and redundant (over represented) reads during sample preparation. Trimmed reads were also checked qualities using FastQC for further analyses. Bowtie2 was used to map trimmed reads to the reference human genome (UCSC hg38). Mapped reads were run through “Peak Calling” step using MACS2. During differential peak analysis, mega-peak sets were provided to standardize group comparison between various group and samples. Furthermore, the analysis insight into the potential occupancy of the protein being ChIPed for at specific genomic loci. Once a mega-peak sets has been derived, sequence reads were counted to know how many reads overlap each interval for each unique sample in counting read step. Then, using the peak locations on chromosomes, nearby-gene information of predicted binding regions were annotated with references databases including Ensembl and UCSC by EdgeR. After differential analysis, the differential peak regions between control and treatment were listed as genomic start and end sites. Annotating with reference databases, most of peak regions could be identified and annotated as various types of region like the Promoter, TSS, Exon, 5’UTR, 3’ UTR, Intronic or Intergenic regions. Those peaks' annotated on promoter region are further performed the motif analysis by MEME-ChIP. Those peaks in treated group significantly higher than control group were used for metabolic pathway and signaling pathway by Ingenuity of pathway analysis (IPA) .
